# Contribution of Protein, Starch, and Fiber Composition to the Prediction of Dough Rheology and Baking Quality in U.S. Hard Red Spring Wheat

**DOI:** 10.3390/foods15040650

**Published:** 2026-02-11

**Authors:** Yun Zhao, Emad Karrar, Jim Peterson, Shahidul Islam

**Affiliations:** 1 Department of Plant Sciences, North Dakota State University, Fargo, ND 58108, USA; yun.zhao@ndsu.edu (Y.Z.); emad.karrar@ndus.edu (E.K.); 2 North Dakota Wheat Commission, Mandan, ND 58554, USA; jpeterso@ndwheat.com

**Keywords:** breadmaking quality, dough rheology, hard red spring wheat, high molecular weight glutenin, multivariate modeling, protein quality, starch composition

## Abstract

Wheat end-product quality results from complex interactions among protein, starch, and fiber, further complicated by genetic and environmental variability, especially in commercial samples composed of multiple varieties from diverse regions. Eighteen composite samples of hard red spring wheat (HRSW) were prepared from 755 field samples to simulate commercial grain blending. These composites were analyzed to evaluate the influence of flour composition on product quality. A wide range of flour compositional properties was analyzed and associated with dough and end-product quality traits, as measured by GlutoPeak, Rapid Visco Analyzer, Farinograph, Extensograph, Alveograph, and loaf baking. The results indicated that dough and bread quality are not determined by protein or gluten content alone, but that protein, starch and fiber composition and structural variations play a crucial role. Flours with higher proportions of high-molecular-weight glutenin (HMW-GS) fractions, particularly those rich in Bx and Ax subunits, exhibited greater dough resistance, mixing strength, and bread volume. In contrast, lower-performing samples were characterized by reduced HMW/LMW, polymeric/monomeric protein ratios, and HMW-Bx content. Multivariate modeling showed strong predictive performance for loaf volume (R^2^ > 0.860) when protein, starch and fiber quality metrics were combined with protein content. These findings provide a data-driven framework for wheat flour classification and optimizing processing formulation.

## 1. Introduction

Several key functional attributes of wheat dough including elasticity, extensibility, and gas retention play a central role in determining bread quality traits such as loaf volume, crumb structure, and overall sensory appeal [1,2]. These properties arise from the formation of a viscoelastic gluten–starch matrix during mixing and hydration. Within this matrix, glutenins contribute strength and elasticity, while gliadins provide extensibility, enabling the dough to stretch and trap fermentation gases effectively. Simultaneously, the size and composition of starch granules influence dough viscosity, swelling, and gelatinization behavior, which are critical to crumb setting during baking [3,4,5]. Recent findings have emphasized the role of glutenin subunit composition and starch damage in modulating dough performance, especially under industrial mixing conditions [6,7].

While protein content has traditionally been used as a key predictor of dough functionality and end-product quality, the influence of starch and dietary fiber is now equally recognized. Starch, which comprises approximately 70–75% of wheat flour, affects water absorption, gelatinization, and pasting behavior, all of which influence bread volume, crumb structure and shelf life. The amylose-to-amylopectin ratio is especially important: higher amylopectin promotes moisture retention and softness, while increased amylose supports a firmer crumb [8,9]. Additionally, starch damage from milling significantly impacts water binding, enzymatic activity, and fermentation, ultimately affecting dough consistency and gas-holding capacity [10,11].

Although refined flour contains a lower proportion of fiber fractions compared to whole wheat, particularly arabinoxylans, these minor components still significantly affect dough properties. Soluble fibers improve water retention and dough viscosity, potentially enhancing gas retention and crumb softness [12,13]. Conversely, insoluble fibers disrupt the gluten network, reducing extensibility and loaf volume [14]. The functional effect of fiber depends on properties such as solubility, molecular weight, polymerization degree, and interactions with gluten and starch. These interactions impact hydration, fermentation stability, and elasticity, contributing to variability in flour performance [15,16].

At the molecular level, gluten functionality is shaped by the composition and interaction of its two primary protein fractions: monomeric gliadins and polymeric glutenins. Gliadins provide viscosity and extensibility, while glutenins, especially the high- (HMW-GS) and low-molecular-weight subunits (LMW-GS) form large, disulfide-linked networks that impart strength and elasticity [17,18]. The glutenin-to-gliadin (Glu/Gli) ratio is a critical determinant of dough strength, with higher values correlating with improved gas retention, mixing stability, and baking performance [19,20]. Another key metric is the ratio of unextractable to extractable polymeric proteins (UPPs/EPPs), which reflects the extent of gluten cross-linking. Higher UPP/EPP values are associated with stronger networks and better dough resilience [21]. The balance between HMW-GS and LMW-GS is also essential, as a higher proportion of HMW-GS enhances dough elasticity and overall bread quality [22]. Despite their widespread use, total protein and wet gluten content often fall short in accurately predicting baking performance. Wheat varieties with similar crude protein levels can exhibit different functional behaviors in baking due to differences in protein fraction composition and polymerization [23,24]. This has led to a challenge in quality evaluation, favoring advanced molecular indicators like Glu/Gli ratio, UPPs/EPPs, and HMW-GS/LMW-GS ratio for better prediction of flour performance.

These traits are influenced by a combination of genetic, environmental, and agronomic factors. Genotypic variation at Glu-1 and Glu-3 loci regulates glutenin subunit expression, which in turn determines dough strength [25,26]. Environmental stress, particularly heat during grain filling, can increase gliadin synthesis at the expense of glutenins, weakening the dough matrix [27]. Additionally, nitrogen application practices such as rate, timing, and form modulate both protein content and composition, affecting baking quality [28]. In commercial milling, the magnitude of the interactive influence of these factors on the flour’s functional properties is further increased due to the blending of diverse wheat varieties grown across different environmental conditions, resulting in unpredictable variability in protein, starch and fiber composition, complicating quality control and limiting the reliability of crude protein content as a standalone predictor of baking performance. Several studies have shown that traditional quality parameters such as protein and wet gluten account for only 40–60% of the variation in end-use baking quality, underscoring the need for more comprehensive and mechanistic models to uncover the remaining source of variabilities [29].

To address this gap, the current study integrates the advanced protein quality indices of Glu/Gli, UPPs, HMW/LMW, and component analysis of gliadins, glutenins, starch, and arabinoxylans to predict the end-product quality of 18 commercial-scale HRS blends covering diverse climatic zones and varieties. The flour compositional markers provide mechanistic insights into gluten formation and starch behavior under processing conditions, enabling a more accurate evaluation of flour quality. By identifying the other influential traits associated with dough rheology and breadmaking potential, this work supports the development of a refined, data-driven framework for flour quality assessment for optimized flour blending, and enhanced quality control in the baking industry.

## 2. Materials and Methods

### 2.1. Materials

The hard red spring (HRS) wheat flour samples analyzed in this study were collected from 755 crop fields/elevators across seven HRS-wheat-producing states: Minnesota, North Dakota, South Dakota, Montana, Washington, Idaho, and Oregon, grown in 2023. The samples were grouped into 18 regional composites according to their geographical position, following the zoning used in the regional crop quality survey (https://f568d15e-c983-41c1-b6d5-1fa22c214e60.filesusr.com/ugd/cd57db_2e255fb3eef64c92bfff7fff21ffb0e0.pdf, accessed on 28 April 2025), conducted by the respective state wheat commissions and U.S. Wheat Associates. For composite sample preparation, all available samples within each region were combined regardless of variety or field location. This approach was designed to simulate the commercial grain supply chain, in which wheat from multiple farms within neighboring regions is mixed during handling at elevators, typically without regard for varietal identification. However, this research did not aim to compare wheat samples based on geographic origin. Therefore, the geographical identities of the composite samples were anonymized using randomly assigned numbers ranging from 1 to 18. Sample preparation and milling were carried out at the HRS Wheat Quality Laboratory, Department of Plant Sciences, North Dakota State University (2023).

### 2.2. Methods

#### 2.2.1. Proximate Composition Analysis

Milling was performed using a Bühler ML-202 laboratory mill (Bühler Group, Gupfenstrasse 5, 9240 Uzwil, Switzerland) under controlled environmental conditions (22–23 °C, 68% relative humidity) to ensure uniformity. Prior to milling, wheat kernels were tempered to 16% moisture content for 16 h, with an additional 0.5% water added 15 min before milling.

Moisture content was determined using the vacuum oven method (AACCI Method 44-40.01), and ash content by dry ashing (AOAC Method 923.03). Crude protein was quantified using the Dumas combustion method (AOAC Method 990.03) via a LECO^®^ FP828P Nitrogen Analyzer (LECO Corporation, 3000 Lakeview Avenue, St. Joseph, MI, USA), applying a nitrogen-to-protein conversion factor of 5.70. Wet gluten and gluten index were measured using the Glutomatic 2200 S (Perten Instruments, Springfield, IL, USA), according to AACCI Method 38-12.02. Starch properties, including total starch, damaged starch, and the amylose-to-amylopectin (AM/AP) ratio, were assessed using Megazyme (Neogen, 620 Lesher Place, Lansing, MI, USA) enzymatic kits (K-TSTA, K-SDAM, and K-AMYL) according to AOAC Method 996.11 and AACCI Method 76-31.01. The arabinoxylan content in the flour was assessed through acid hydrolysis and alditol acetate derivatization, as outlined in the procedure by [30]. Subsequently, the derivatized samples were examined using gas chromatography with flame ionization detection, following the method described by [31]. All analyses were conducted in duplicate.

#### 2.2.2. Protein Fractionation and Quantification

To evaluate gluten quality, the SDS-extractable and unextractable polymeric proteins (EPPs and UPPs) were extracted from 60 mg of flour using 0.05 M phosphate buffer (pH 6.9) containing 0.5% SDS, following [32]. Gliadin and glutenin fractions were sequentially extracted as described by [33]: albumins and globulins were removed using 55% isopropanol; gliadins were extracted with 70% ethanol; and glutenins with 0.08 M Tris-HCl (pH 8.0) containing 50% isopropanol, 1% DTT, and 1.4% 4-vinylpyridine. All extracts were filtered through 0.45 µm PVDF (Polyvinylidene Fluoride) membranes prior to analysis.

Size-exclusion HPLC (SE-HPLC) was performed using an Agilent 1100 LC system (Agilent Technologies, 5301 Stevens Creek Blvd, Santa Clara, CA, USA) with a Bio SEC-5 column (4.6 × 300 mm, 500 Å) at 25 °C. A 50:50 gradient of water (0.1% TFA) and acetonitrile (0.1% TFA) was run at a constant 0.35 mL/min over 20 min, with detection at 210 nm. Reverse-phase HPLC (RP-HPLC) for glutenin and gliadin analysis used a Zorbax 300SB-C18 column (Agilent Technologies, 5301 Stevens Creek Blvd, Santa Clara, CA, USA) (4.6 × 250 mm, 5 µm, 300 Å) at 55 °C, with a 21–47% acetonitrile gradient over 45 min at 1.0 mL/min. The data were processed with OpenLab software V3.6 (Agilent Technologies, 5301 Stevens Creek Blvd, Santa Clara, CA, USA). Derived indices included the polymeric-to-monomeric protein ratio (P/M), glutenin-to-gliadin ratio (Glu/Gli), UPP percentage, gliadin subclass distribution (ω-, α/β-, and γ-gliadins), and the HMW-GS/LMW-GS ratio.

#### 2.2.3. Starch Pasting Properties Analysis

Pasting behavior was assessed using a Rapid Visco Analyzer (RVA; Newport Scientific Inc., Jessup, MD, USA) per AACCI Method 76-21.01 with slight modifications. A 3.5 g flour sample (14% moisture basis) was mixed with 25 mL distilled water and subjected to a heating and cooling cycle: 50 °C hold, heating to 95 °C, 2.5 min hold, cooling to 50 °C, and a final hold. Parameters recorded included pasting temperature, peak viscosity, breakdown, final viscosity, and setback.

#### 2.2.4. Gluten Strength and Dough Rheological Analysis

The GlutoPeak test was conducted to obtain gluten-strength-related parameters using a GlutoPeak device (Brabender, Duisburg, Germany), according to [34]. An aliquot of 8.5 g of sample was dispersed in 9.5 g of 0.5 mol∙L^−1^ CaCl_2_, scaling both solvent and flour weight on a 14% flour moisture basis in order to keep the liquid-to-solid ratio constant. Sample temperature was maintained at 34 °C. The paddle was set to rotate at 1900 rpm and the test was carried out for 7 min. The peak maximum time (s), maximum torque (BU), and aggregation energy (cm^2^) were automatically determined by the software provided by the manufacturer.

##### Farinograph

Mixing characteristics were determined using a Farinograph-E (C.W. Brabender, Duisburg, Germany) equipped with a 50 g mixing bowl, according to AACCI Method 54-21.02. Tests were conducted at 30 ± 0.2 °C, with a constant mixing speed of 63 rpm. Water addition was adjusted to obtain a dough consistency of 500 ± 10 Brabender Units (BU). The parameters were generated automatically by the Farinograph software, V2.1.6, including water absorption capacity (WAC), dough development time, dough stability, mixing tolerance index (MTI), and the overall quality number, all of which provide insight into the hydration behavior and mechanical strength of the dough during mixing.

##### Extensograph

Dough extensibility properties were assessed using an Extensograph-E (Brabender, Germany) following AACCI Method 54-10.01. Doughs were prepared using the optimum water absorption obtained from Farinograph tests and allowed to rest for 45 min at 30 ± 1 °C and 85% relative humidity before testing. The dough was stretched at a constant speed of 45 mm/min until rupture. Measurements were performed after 45, 90, and 135 min of proofing to assess dough development over time. The key parameters of resistance to extension, maximum extensibility, and energy (area under the curve) were recorded to evaluate the viscoelastic strength and extensibility of the gluten network.

##### Alveograph

The Alveograph test was conducted based on AACCI Method 54-30.02 to simulate dough behavior during fermentation and gas expansion. A dough sheet was prepared by mixing 250 g of flour with the appropriate amount of water (to achieve 50% hydration) and 2.5% NaCl. The dough rested for 20 ± 2 min at 25 ± 1 °C and a relative humidity of 85% before testing. Air was blown beneath the dough sheet until rupture to record the overpressure curve. This method yielded critical rheological indicators: P (tenacity or resistance to deformation), L (extensibility), P/L ratio (indicating the balance between strength and extensibility), and W (baking strength, represented by the area under the curve).

Together, these rheological tests provide a comprehensive profile of flour quality and dough performance relevant to baking applications.

#### 2.2.5. Breadmaking Quality Evaluation

Bread quality was evaluated using 100 g pup loaf tests based on AACCI Method 10-09.01, with adjustments for laboratory settings. Fermentation lasted 2 h using instant dry yeast and fungal amylase instead of compressed yeast and malt powder. A 10% ammonium phosphate solution (1 mL/100 g flour) was added to enhance yeast activity. Loaf volume was measured using the rapeseed displacement method (AACCI Method 10-05.01), and crumb structure and texture were assessed per AACCI Method 10-12.01.

#### 2.2.6. Statistical Analysis

Analysis of variance and significant differences among means, correlation analysis, and boosted tree prediction were tested by JMP Pro 17 (JMP Statistical Discovery LLC). Differences were considered significant when the probability value p was lower than 0.05. A least significant difference (LSD) test with a 5% significance level was used to declare differences. Data were presented as the means of two technical replicates of each composite samples, with standard errors averaged. MetaboAnalyst 5.0 (https://www.metaboanalyst.ca/home.xhtml (accessed on 28 April 2025)) was used for the analysis of heatmap and variable importance in the projection (VIP), and to analyze the relationships existing between different traits.

## 3. Results and Discussion

### 3.1. Multivariate Diversity Analysis of Flour Compositional and Molecular Properties

#### 3.1.1. Flour Composition

The samples exhibited considerable variation across key flour compositional parameters including protein, starch, ash (mineral) and fiber (arabinoxylans) content (Table 1). For example, grain protein content (GPC) ranged from 14.67% to 16.33%, which indicates a considerable variation that may influence the flour functional properties and baking quality. Similarly, ash content demonstrated a sizable difference, ranging from 0.44% to 0.54%, reflecting differences in flour mineral content. However, this range agreed with the ash content range of HRS wheat (0.46–0.76%) [35], which is higher than the ash content of hard red winter wheats [36]. Samples with a higher ash content likely contained higher proportions of fiber and minerals that increase water absorption and affect dough properties [37].

The total starch content did not show significant differences among the 18 composite samples, which suggests the stability of this trait across different HRS growing regions. Typically, HRS wheat has lower total starch than soft wheat [29]. A significant variation in total arabinoxylan content was observed among the 18 samples, ranging from 14.45 to 27.48 mg/g. This variation may partially reflect regional differences in cultivar composition with different AX content genotypes. Environmental conditions such as temperature, precipitation, and soil fertility could also cause differences in AX accumulation among these samples [38]. Arabinoxylans are major components of wheat dietary fiber. Apart from their nutritional value, their strong water retention capacity also affects wheat functionality during the breadmaking process [39]. The regional variation observed here may reflect long-term breeding emphasis and environmental adaptation in the northern plains HRSW production system.

#### 3.1.2. Protein Compositional and Molecular Variations

Variations were observed for the protein polymer compositional parameters (Table 2) and the protein type compositional parameters (Table 3).

Wet gluten content varied between 31.12% and 36.80%, reflecting significant differences in both protein quantity and hydration capacity. These values are consistent with the established range for the HRS wheat class [36,40]. The wide range in gluten index of 81.31 to 96.32 indicates notable variation in gluten strength, likely influenced by differences in polymerization levels and glutenin subunit composition.

Results from the SE-HPLC analyses demonstrated that the UPP% ranged between 45.98% and 61.16%, while for the majority of samples, 13 samples out of 18 had a UPP% over 59%, reflecting the high-gluten properties of HRS wheats. High UPP% was reported to correlate with good protein and breadmaking quality [29,36]. Variations were also found on polymeric protein% (28.68–38.83%) and polymeric/monomeric ratio (0.40–0.64). Polymeric proteins include the HMW and LMW glutenins, both actively involved in the dough rheology process, contributing to the bread’s strength and mixing tolerance [41].

On the other hand, Glu/Gli ratio ranged from 0.57 to 0.86. The ratio of Glu/Gli affects the functional properties of the dough and the quality of the final products. A higher ratio of glutenin proteins improves dough stability [42]. In addition, different Glu/Gli ratios also cause rearrangements of hydrogen bonds and can thus determine the gelatinization properties of the starch and the starch–gluten matrix [43]. High-resolution RP-HPLC analyses demonstrated variation in the HMW-to-LMW glutenin ratios (HMW/LMW) and the HMW%, ranging from 0.69 to 0.87 and 40.88% to 46.50%, respectively. An elevated HMW/LMW ratio enhances dough elasticity and gas retention by increasing the number of inter-chain disulfide linkages within the gluten matrix, resulting in a stronger gluten network that can better withstand the pressure of fermentative gas expansion during proofing and baking [44].

As for the protein compositional differences on a more detailed level, both the gliadin and glutenin subunits were analyzed and they demonstrated significant variation between the 18 composite samples (Table 3). Gliadins affect the dough extensibility; a larger amount of gliadins increases dough extensibility at the expense of the gluten network strength [45]. The proportion of ω-gliadins, α/β-gliadins, and γ-gliadins ranged from 9.59–12.42%, 37.95–46.68%, and 47.52–51.91%, respectively. Among them, ω-gliadins are widely considered to negatively affect breadmaking quality because they lack the cysteine residues necessary for forming disulfide bonds, preventing them from participating in the gluten polymer network [46,47]. Variations were also observed for the HMW-GS compositions. Each Glu-1 locus on the long arm of the group 1 chromosomes (1A, 1B, and 1D) encodes one or two HMW-GSs, designated as x-type or y-type based on their relative electrophoretic mobility (ax, bx, by, dx, dy). These subunits differ in size and cysteine content, which determines their ability to form inter-chain disulfide bonds within the glutenin macropolymer [48]. The ax and dy HMW-GSs were found in a lower amount (4.74–5.96% and 3.25–4.72%) than the by + dx and bx HMW-GSs (11.88–15.29% and 7.57–12.54%). HMW-GSs are considered to have a more important role than LMW-GSs and gliadins in maintaining dough strength [49]. Changes in subunit composition will change the secondary structure of the HMW-GSs, including the contents and ratios of the α-helices and β-sheets. Abundant bx and ax subunits were reported to promote dough strength and loaf volume [50].

#### 3.1.3. Starch Compositional Variations

Starch compositional parameters including the amylose content, amylopectin content, and amylose/amylopectin (AM/AP) ratio were investigated in this study, all exhibiting significant variations among the 18 samples (Table 4). Amylose and amylopectin content ranged from 21.65% to 24.74% and 74.27% to 80.97%, respectively. While AM/AP ranged from 0.24 to 0.35. As reported by [51], this ratio significantly impacts starch gelatinization behavior, crumb firmness and shelf life through retrogradation effects. Amylose limits water absorption and the swelling of starch granules, while amylopectin promotes swelling ability and maintains granule integrity. These parameters are the major source of variation for wheat starch pasting properties [52].

The mechanical breakdown of the endosperm and heat during milling causes damage to starch and protein structures, which can lead to an increase in flour hydration capacity [53]. The cavity formation and exposure of amylose and amylopectin hydroxyl groups in damaged starch is the main reason for the increase in water absorption capacity [10]. In this study, starch damage ranged from 5.90% to 7.00%, which is considered moderate and suitable for breadmaking. Excessive starch damage, generally over 8%, will potentially disrupt gluten network formation, likely through competitive hydration effects [54].

#### 3.1.4. Fiber (Arabinoxylan) Compositional Variations

Analysis of arabinose and xylose content across the 18 HRSW composite samples revealed considerable natural variation in arabinoxylan (AX) composition (Table 4). Arabinose content ranged from 5.95 to 12.05 mg/g and xylose content ranged from 8.50 to 16.95 mg/g, with the arabinose-to-xylose (A:X) ratios varying between 0.62 and 0.91. Lower A:X ratios, indicate less-substituted, more linear AX chains, which are known to enhance gluten fiber interactions [12]. By contrast, variations in arabinose content, which reflects the branching degree of arabinoxylans, may exert a greater influence on water absorption capacity and thus dough gas retention due to changes in solubility and hydration behavior [55].

### 3.2. Multivariate Diversity Analysis of Functional Behavior in Processing

#### 3.2.1. Flour Pasting and Gelatinization Variations

Pasting properties are crucial in defining the functional performance of wheat flour during thermal processing. In this study, the 18 HRSW composite samples exhibited considerable variation in pasting characteristics (Table S1). RVA analysis showed peak viscosity values ranging from 1432.5 to 2312.5 cP, with breakdown values between 280 and 687 cP, reflecting differences in starch granule integrity and its susceptibility to enzymatic breakdown. These variations in RVA are likely due to the structural variations in the starch crystal system which influence enzyme accessibility and hydrolysis during dough fermentation and baking. As reported by [56], higher breakdown values indicate greater starch fragility, whereas lower values, such as that of sample no. 18 (280 cP), suggest a higher amount of heat-stable intact starch, which is a desirable trait for products requiring thermal stability. Pasting temperatures varied from 68.98 °C to 94.48 °C, further differentiating the samples. Those with lower pasting temperatures may promote earlier starch gelatinization and softer textures, while samples with higher pasting temperatures tend to exhibit greater thermal resilience, consistent with findings by [57] in flour blends used for steamed bread and buns.

#### 3.2.2. Gluten Strength and Aggregation Behavior Variations

GlutoPeak testing provides a rapid means to evaluate gluten aggregation properties, offering valuable insight into the structural behavior of wheat proteins under mechanical stress during processing. In this study, GlutoPeak analysis of 18 HRSW flour samples (Table S2) revealed notable differences in gluten strength and development, highlighting their functional diversity and suitability for various end-use applications. Peak maximum time ranged from 81 to 138 s, while maximum torque values varied between 54.00 and 60.67 Brabender Units (BU). Generally, faster aggregation (shorter times) was associated with weaker gluten networks, indicative of more stable gluten development. High GlutoPeak torque and aggregation energy correlates with superior mixing tolerance and baking stability [58].

#### 3.2.3. Variations in Water Absorption and Dough Mixing Properties

Understanding the rheological properties of wheat flour is fundamental for optimizing performance across a range of bakery products. Different instruments including Farinographs, Extensographs, and Alveographs are commonly used to assess the different aspects of dough rheological properties. A Farinograph can be considered a Searle-type viscometer that detects dough mixing behavior including water absorption, optimum mixing time and mixing tolerances. Farinograph parameters are considered classic indicators of dough physical properties [59,60,61]. In this study, a Farinograph was used to compare the water absorption and dough mixing properties of the 18 composite samples. Variations were found among these samples (Table S3). Water absorption capacity (WAC) has proved to be a vital trait for the evaluation of breadmaking quality and is generally influenced by several flour compositional properties including protein, gluten, damaged starch, and fiber content [62]. Higher water absorption capacity is desirable for good breadmaking performance and is also a commercially important parameter. The WAC among the 18 samples varied from 61.35% to 65.15%. These values are consistent with the typically high WAC observed in HRSW, which generally exceeds 60%, whereas soft-wheat classes are usually characterized by a lower WAC. Another important Farinograph parameter, dough stability, reflects the flour tolerance to mixing. This trait is strongly related to gluten strength, where higher values indicate stronger gluten [63]. In this study, stability varied significantly from 8.55 to 19.20 min. The other parameter, mixing tolerance index (MTI), indicates the flour’s ability to handle the over-mixing, the lower the MTI value, the better the dough’s resistance to mechanical damage. Among the 18 samples, MTI ranged from 14 to 29.5, classifying most samples as medium-to-strong doughs according to ICC standards.

#### 3.2.4. Variations in Dough Strength and Stretching Properties

Dough strength, resistance and extensibility against applied force or energy are also important rheological properties. Here, the 18 HRSW samples were assessed using an Extensograph and an Alveograph to evaluate the physical dough properties which have a strong influence on baking potential. Alveograph data (Table S4) quantified biaxial dough expansion, a critical factor in dough deformation under gas pressure. The P (tenacity) values ranged from 72.67 to 108 mm H_2_O, and baking strength (W) spanned 319.33 to 480.67 × 10^−4^ J. High-performing samples, such as 1 and 11, combined elevated P and W values, reflecting desirable dough resistance and elasticity. These results support [64], who reported that optimal breadmaking flours achieve a P/L ratio near 0.8, balancing dough strength and extensibility as exemplified by sample 11 (P/L = 0.99). An Extensograph measures the stretchability of dough. The parameters obtained through Extensograph analysis are an indirect reflection of dough behaviors at further stages of bread production [65,66]. Breadmaking flours require dough with a moderate resistance but a high extensibility [67]. Extensograph tests conducted at 45 and 135 min showed significant variation in dough extensibility (cm) and resistance (U), reflecting structural changes during resting. Extensibility at 45 and 135 min ranged from 15.40 to 18.48 cm, and 11.80 to 16.63 cm, respectively. Resistance at 45 and 135 min ranged from 405.50 to 702.00 U and 706.75 to 1366.50 U, respectively. Samples that maintain high resistance and extensibility indicate a strong protein cross-linking and backbone integrity [68].

#### 3.2.5. Variations in the Baking Quality

Finally, the baking performance of the 18 HRSW composite samples was evaluated to provide an integrated perspective on how flour composition and dough rheology influence bread quality (Table 5). Baking trials confirmed the ultimate functional impact of compositional variability, with loaf volumes ranging from 840 to 1062.5 mL. Samples 11, 12, and 13 stood out, delivering superior baking outcomes in terms of loaf volume, symmetry, and crumb structure. These high-performing samples consistently exhibited a high glu index (>93), a moderate arabinoxylan content (20–23 mg/g), and favorable pasting and gluten aggregation profiles as indicated by RVA and GlutoPeak analyses. These results support integrative quality frameworks such as those proposed by [69], which emphasize that bread quality arises from the synergistic interaction of protein, starch, and fiber components rather than any single trait. In conclusion, the phenotypic variation observed across all functional traits underscores the compositional complexity and versatile end-use potential of HRSW flours.

This study identified a broader HMW/LMW glutenin ratio range (63–86%) than typically reported for commercial cultivars (70–80%), alongside greater diversity in GlutoPeak torque and aggregation energy, especially in gliadin-rich samples. Moreover, the wide range in pasting temperatures and setback values reflects substantial differences in starch structure and thermal behavior. Collectively, these findings highlight the importance of a comprehensive, multi-trait analytical approach to pinpoint key dough rheological and end-product quality determinants in HRSW. Such a framework enhances selection criteria for breeding and milling programs while informing predictive formulation strategies tailored to specific baking applications.

### 3.3. Trait Association Analysis

**Figure 1:**
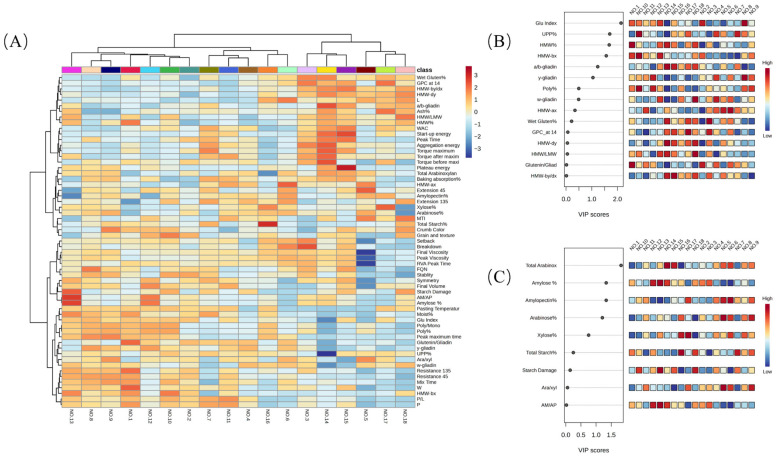
Hierarchical clustering of all variables and selected variable importance in the projection of the samples in this study. (**A**) Hierarchical clustering heatmap of all variables; (**B**) importance in the projection of protein-related traits; (**C**) importance in the projection of starch- and fiber-related traits.

Figure 1 presents a comprehensive multivariate evaluation of the compositional factors, pasting, dough, and bread properties of the HRS wheats in this study through hierarchical clustering. Variable importance in projection (VIP) scores of the protein characteristics (Panel B) and starch and fiber characteristics (Panel C) derived from partial least squares (PLS) regression explored the key performance traits based on their rank of predictive value. These analyses collectively emphasize the pivotal roles of gluten subunit composition and starch structure in determining dough strength and loaf volume. The heatmap in Panel A illustrates that flour performance is strongly influenced by the protein content. However, it appears that the intricate interactions among the specific gluten components, starch characteristics, and fiber components also influences the dough and bread quality. For examples, samples no. 1, 11, and 13 exemplified high-performance flours, characterized by high tenacity (P ≥ 108 mm H_2_O), high baking strength (W ≥ 480 × 10^−4^ J), strong dough elasticity (resistance at 45 min ≥ 638 U, resistance ≥ 1073 U), and loaf volumes exceeding 890 cm^3^. These traits coincided with strong expression of HMW glutenin subunits, particularly HMW-bx and HMW-ax, confirming the robust gluten polymerization essential for dough stability and gas retention [22,70]. Conversely, samples 6 and 14 grouped among the weaker flours, marked by high extensibility (Extension ≥ 15.90 cm) but lower resistance and loaf volume, with gluten profiles demonstrated low HMW/LWM, low poly/mono ratios, and a low HMW-bx. This supports reports by [5,19] indicating that enhanced extensibility can compromise the network integrity traits favorable for products like flatbreads and laminated doughs. Sample 13 exhibited a balanced profile, combining moderate extensibility, high resistance, and loaf volume (~880 cm^3^), likely due to its moderate AM/AP ratio, balanced gluten composition, and slightly elevated starch damage (~6.6%), which improved water absorption and dough handling [71].

The VIP scores in Panel B identify gluten index, UPP%, HMW%, and HMW-bx as the most influential protein-related predictors, supporting their utility in functional flour screening [72]. While Panel C indicates that total arabinoxylans content, amylose%, and arabinose% are the most influential factors regarding starch and fiber, which is consistent with the important function of arabinoxylans’ water-holding capacity during breadmaking and bread storage [73] and the significant effects of starch composition on bread quality and shelf-life [74]. Altogether, these results suggest that functional quality in HRSW flour emerges from a synergistic balance of gluten subunit diversity and starch architecture, providing clear markers for genotype selection, flour blending, and targeted product development.

Table S5 demonstrated the correlation analysis between the protein, starch, and fiber compositional factors and the flour pasting, dough rheology and breadmaking qualities. Bread volume is affected by multiple factors; it showed positive correlations with gluten index (r = 0.549), AM/AP (r = 0.423), and γ-gliadin content (r = 0.348), suggesting protein quantity, protein quality, and starch composition jointly influence the bread quality.

Protein quantity has been identified as the major factor for dough and bread quality determination in this study. The correlation analysis revealed that it possibly influences bread quality by affecting gluten strength and aggregation, dough mixing, and stretching properties, reflected by its positive correlation with Glutopeak toque before maximum (r = 0.401), aggregation energy (0.402), Farinograph WAC (r = 0.416), and Alveograph L (r = 0.386). Furthermore, different fractions of protein influence bread quality in different manners. Gliadins demonstrated both negative and positive effects on dough and bread quality. ω-gliadins showed a negative correlation with Glutopeak torque maximum (r = −0.577), Farinograph WAC (r = −0.600) and PT (r = −0.632). α/β-gliadins showed a positive correlation with Glutopeak torque maximum (r = 0.545) and Farinograph WAC (r = 0.441). γ-gliadins showed a positive correlation with Glutopeak Peak maximum time (r = 0.446), and Extensograph resistance at 45 min (r = 0.360).

Glutenin subunit compositions were widely accepted as major determinators of gluten functionality [48,75,76]. In this study, glutenin subunits (Ax, Bx, By, Dx and Dy) demonstrate strong influence on dough and bread quality parameters, mainly by affecting gluten strength and stretching properties. Ax subunit of HMW glutenin showed positive correlation with dough extensibility at 135 min (r = 0.496). On the other hand, subunit Bx showed positive correlation with Glutopeak peak maximum time (r = 0.523), Extensograph resistance at 45 min (r = 0.682) and 135 min (r = 0.612), Alveograph P (r = 0.384), and P/L (r = 0.388). Two other subunits which appeared combinedly in HPLC separation, By and Dx, showed a positive correlation with Glutopeak torque maximum (r = 0.460), Farinograph WAC (r = 0.596), and Alveograph L (r = 0.470), G (r = 0.477). The other subunit Dy showed a positive correlation with Farinograph WAC (r = 0.342), and Alveograph L (r = 0.436), and G (r = 0.440).

Besides the proportion of individual protein components, the relative proportion of protein classes can also affect dough and bread quality. A lower amount of HMW-GSs is responsible for the decay of dough strength [77]. In this study, poly/mono ratio showed a positive correlation with Glutopeak maximum time (r = 0.794), Farinograph stability (r = 0.454), and Extensograph resistance, both at 45 (r = 0.564) and 135 min (r = 0.463). Glu/Gli ratio showed positive correlation with Farinograph stability (r = 0.403), Extensograph resistance at 45 (r = 0.434) and 135 min (r = 0.387), Alveograph P (r = 0.534), and P/L (r = 0.472). Similar correlations were reported previously [78]. These results highlight the critical role of glutenin polymer strength, rather than total protein, in conferring dough viscoelasticity and fermentation tolerance in HRS wheat, representative of the commercial supply chain.

Apart from protein, starch properties also played an important role in dough and bread quality parameters. Starch damage showed a positive correlation with Farinograph WAC (r = 0.350), which is consistent with previous reports [10,53]. Amylose content showed a positive correlation with Alveograph P/L (r = 0.337). Amylopectin content showed a positive correlation with Extensograph Extension at 45 (r = 0.352) and 135 min (r = 0.406), and Alveograph L (r = 0.429) and G (r = 0.435). Our findings indicate that samples with moderate AM/AP ratios (21–22%) performed better in baking tests compared to those at the extremes, corroborating [74], who observed that high amylose content tends to increase crumb firmness, whereas low amylose levels weaken crumb structure and reduce shelf stability.

Arabinoxylan and ash content are important flour compositional factors affecting water absorption [79]. Total arabinoxylan content showed a positive correlation with baking water absorption (r = 0.338). Importantly, samples with lower A:X ratios demonstrated superior loaf volume and improved dough-handling properties, aligning with the findings of [80], who emphasized the role of AX branching in influencing baking performance and fermentation stability. Ash content showed a positive correlation with WAC (r = 0.570), and Glutopeak torque maximum (r = 0.344), underscoring the relationship between mineral content and flour hydration properties.

Mechanistically, high proportions of HMW glutenin subunits (Bx, Ax) enhance disulfide cross-linking, increasing network elasticity, whereas high gliadin or soluble arabinoxylan levels promote viscosity but reduce stability. Similarly, starch with moderate amylose content enhances gas cell support during baking by forming partial gels that reinforce the gluten matrix. These interactions demonstrate the integrated biochemical basis for dough behavior observed in this study.

### 3.4. Prediction Modeling

The use of boosted regression tree models provided a powerful and interpretable approach to evaluate how flour compositional traits affect two key quality indicators: bread loaf volume and water absorption capacity (WAC) (Table 6). These models demonstrated that integrating protein, starch, and fiber variables yields more accurate predictions than considering either group alone. To improve model interpretability, simplified linear regression equations were generated with cross-validation R squares relative to the boosted tree outputs (Table S6). For loaf volume, one of the top-performing models combined grain protein content (GPC at 14%), wet gluten, and RP-HPLC composition (R^2^ = 0.861). The simplified linear regression equation of this model is expressed as:

Final volume = −872.36 − 22.59 × ω-gliadins + 6.45 × α/β-gliadins + 16.78 × γ-gliadins − 16.48 × HMW-dy − 1.84 × HMW-by/dx + 19.28 × HMW-bx − 23.84 × HMW-ax + 58.55 × HMW% − 1952.42 × HMW/LMW

The linear model achieved an R^2^ of 0.74 relative to the boosted tree outputs, indicating that approximately 86% of the predictive information from the non-linear model was retained. The other top-performing model combined GPC, wet gluten, and SE-HPLC composition (R^2^ = 0.831), emphasizing the importance of both protein quantity and quality. Notably, high-molecular-weight glutenin subunits, particularly Bx and Ax, appeared to be major contributors due to their role in promoting gluten polymerization and forming the elastic networks critical for gas retention during fermentation [75,81]. Even a model relying solely on RP-HPLC glutenin and gliadin traits achieved strong accuracy (R^2^ = 0.842), highlighting their predictive value. In contrast, models based only on protein quantity (R^2^ = 0.667) or starch traits (R^2^ = 0.634), or fiber traits (R^2^ = 0.711) showed less-strong prediction, underscoring that neither class fully captures the complexity of loaf development. These results indicate that, although protein content plays the most important role, incorporating protein quality attributes into the prediction model could further explain the behavior of high-protein-class flour. Furthermore, incorporating starch traits into protein-based models improved predictions (R^2^ = 0.785), similarly to incorporating fiber traits (R^2^ = 0.869), reflecting the synergistic interplay among gluten structure, fiber and starch gelatinization during baking [52]. Models focusing exclusively on RP-HPLC gliadins/glutenins or SE-HPLC polymeric protein metrics were weaker than those combined with protein quantity, suggesting these traits are more relevant to dough extensibility and mixing properties than to loaf volume [82]. Overall, these findings confirm that a multi-trait, integrative modeling approach is essential for comprehensively understanding and optimizing flour functionality. This offers millers and bakers valuable data-driven tools for quality control, product formulation, and innovation.

## 4. Conclusions

This study offers a comprehensive and integrative assessment of supply chain-representative HRS wheat flours, connecting molecular composition to functional performance through detailed profiling, rheological testing, baking trials, and predictive modeling. By analyzing gluten protein compositions, starch characteristics, and fiber components, we demonstrated that while protein content plays the crucial role in determining dough rheological behavior and bread quality, the complex interplay between gluten structure and other chemical components can improve its predictability. Flours with higher polymeric glutenin ratios, particularly those rich in Bx and Ax subunits consistently exhibited enhanced dough resistance, mixing strength, and bread volume, underscoring the critical role of gluten polymerization in gas retention and loaf formation. Starch composition also emerged as a key factor influencing water absorption, pasting behavior, and crumb quality, especially through its interaction with strong protein networks. With its high water-holding ability, arabinoxylan has a marked impact on bread volume and quality and is an essential factor to consider. These findings highlight that while protein content remains a key determinant of wheat functionality, integrating compositional markers such as UPP%, HMW/LMW ratio, AM/AP ratio, and total arabinoxylan provides a more reliable basis for predicting end-use performance. For breadmaking, flours with a balanced P/L (≈1), high UPP (>55%), and moderate AM/AP (≈0.3) ratio yield optimal loaf volume and crumb structure. This compositional framework offers valuable guidance for tailored flour blending and classification strategies in bread product development. Future research should aim to expand the scope to include a broader range of end-product types and wheat varieties, thereby enabling the development of product-specific prediction models.

## Figures and Tables

**Table 1:** Flour compositional characteristics of the 18 HRS composite samples designed to mimic commercial grain samples entering supply chain entry points.

Sample ID	Grain Protein Content %	Ash Content %	Moisture Content %	Total Starch %	Total Arabinoxylans (mg/g)
No.1	15.16 ± 0.01 ^ef^	0.47 ± 0.02 ^f^	14.43 ± 0.01 ^ab^	67.81 ± 0.47 ^bc^	17.45 ± 0.92 ^ef^
No.2	15.16 ± 0.06 ^ef^	0.49 ± 0.00 ^ef^	13.91 ± 0.01 ^cd^	67.04 ± 0.65 ^bc^	23.30 ± 1.70 ^abcd^
No.3	16.27 ± 0.06 ^a^	0.49 ± 0.00 ^ef^	13.31 ± 0.03 ^fg^	65.90 ± 0.77 ^c^	22.00 ± 4.67 ^bcd^
No.4	15.28 ± 0.10 ^de^	0.48 ± 0.00 ^f^	13.22 ± 0.01 ^fg^	67.15 ± 2.10 ^bc^	24.45 ± 2.19 ^abc^
No.5	15.03 ± 0.04 ^fg^	0.47 ± 0.02 ^f^	12.78 ± 0.01 ^h^	66.94 ± 2.08 ^bc^	25.25 ± 0.35 ^ab^
No.6	15.60 ± 0.17 ^c^	0.49 ± 0.01 ^ef^	13.19 ± 0.01 ^fg^	66.87 ± 0.69 ^bc^	25.35 ± 2.05 ^ab^
No.7	14.75 ± 0.02 ^h^	0.49 ± 0.01 ^def^	14.37 ± 0.04 ^ab^	68.72 ± 0.65 ^ab^	19.75 ± 0.07 ^de^
No.8	15.04 ± 0.06 ^fg^	0.44 ± 0.01 ^g^	14.18 ± 0.01 ^bc^	67.38 ± 0.88 ^bc^	24.65 ± 0.64 ^abc^
No.9	14.76 ± 0.07 ^h^	0.44 ± 0.01 ^g^	14.00 ± 0.01 ^c^	68.19 ± 2.53 ^abc^	25.15 ± 1.20 ^ab^
No.10	15.01 ± 0.06 ^g^	0.48 ± 0.01 ^f^	13.62 ± 0.01 ^e^	67.16 ± 0.69 ^bc^	20.50 ± 0.28 ^cde^
No.11	14.67 ± 0.00 ^h^	0.49 ± 0.01 ^ef^	13.65 ± 0.01 ^de^	66.43 ± 1.87 ^bc^	23.75 ± 1.34 ^abcd^
No.12	15.09 ± 0.07 ^fg^	0.49 ± 0.01 ^ef^	13.71 ± 0.0 2 ^de^	67.58 ± 0.31 ^bc^	20.90 ± 0.71 ^bcde^
No.13	15.15 ± 0.01 ^efg^	0.51 ± 0.01 ^bcd^	14.53 ± 0.0 ^a^	66.79 ± 0.04 ^bc^	23.80 ± 1.70 ^abcd^
No.14	15.90 ± 0.11 ^b^	0.53 ± 0.02 ^ab^	12.76 ± 0.01 ^h^	66.56 ± 0.68 ^bc^	27.48 ± 1.67 ^a^
No.15	16.02 ± 0.10 ^b^	0.51 ± 0.01 ^cde^	13.10 ± 0.01 ^g^	66.48 ± 0.06 ^bc^	27.05 ± 5.59 ^a^
No.16	15.33 ± 0.02 ^d^	0.49 ± 0.02 ^ef^	13.93 ± 0.01 ^cd^	70.70 ± 0.24 ^a^	14.45 ± 0.07 ^f^
No.17	16.33 ± 0.04 ^a^	0.54 ± 0.00 ^a^	13.43 ± 0.01 ^ef^	67.03 ± 1.35 ^bc^	22.50 ± 0.14 ^bcd^
No.18	15.42 ± 0.07 ^d^	0.52 ± 0.00 ^bc^	13.19 ± 0.02 ^fg^	68.65 ± 1.24 ^ab^	21.14 ± 2.32 ^bcde^

Data represent mean value ± standard error. Values with different superscripts in a column differ significantly (*p* < 0.05).

**Table 2:** Protein compositional characteristics of the 18 HRS composite samples.

Sample ID	Glu Index	Wet Gluten Content %	UPP %	Polymeric Proteins %	Polymeric/ Monomeric	Glutenin/ Gliadin	HMW %	HMW/LMW
No.1	95.38 ± 0.89 ^a^	34.36 ± 0.18 ^cde^	56.74 ± 5.13 ^b^	37.41 ± 2.87 ^ab^	0.60 ± 0.07 ^a^	0.86 ± 0.01 ^a^	44.41 ± 4.07 ^abcde^	0.80 ± 0.13 ^abcde^
No.2	95.57 ± 0.79 ^a^	31.12 ± 0.02 ^h^	59.05 ± 1.66 ^ab^	32.95 ± 0.02 ^def^	0.49 ± 0.00 ^cdef^	0.79 ± 0.00 ^b^	41.63 ± 0.14 ^cde^	0.71 ± 0.00 ^cde^
No.3	88.35 ± 3.59 ^ef^	36.80 ± 0.26 ^a^	56.34 ± 0.32 ^b^	35.45 ± 0.22 ^abcde^	0.55 ± 0.01 ^abcd^	0.71 ± 0.01 ^de^	44.61 ± 0.49 ^abcde^	0.81 ± 0.02 ^abcde^
No.4	89.51 ± 4.31 ^cdef^	33.48 ± 0.45 ^f^	60.13 ± 2.02 ^ab^	33.48 ± 0.72 ^bcdef^	0.50 ± 0.02 ^bcde^	0.78 ± 0.01 ^b^	40.88 ± 1.87 ^e^	0.69 ± 0.05 ^e^
No.5	85.07 ± 1.24 ^fg^	34.61 ± 0.39 ^cd^	59.76 ± 0.99 ^ab^	35.16 ± 2.47 ^abcde^	0.54 ± 0.06 ^abcd^	0.65 ± 0.00 ^gh^	43.59 ± 2.26 ^abcde^	0.77 ± 0.07 ^abcde^
No.6	90.67 ± 0.89 ^bcde^	34.80 ± 0.22 ^cd^	58.47 ± 2.92 ^ab^	33.26 ± 0.39 ^cdef^	0.50 ± 0.01 ^bcdef^	0.78 ± 0.01 ^b^	42.54 ± 1.99 ^bcde^	0.74 ± 0.06 ^bcde^
No.7	90.02 ± 1.25 ^cde^	34.26 ± 0.53 ^de^	60.94 ± 0.06 ^a^	32.25 ± 0.15 ^efg^	0.48 ± 0.00 ^def^	0.76 ± 0.00 ^bc^	42.46 ± 0.08 ^bcde^	0.74 ± 0.00 ^bcde^
No.8	96.32 ± 0.80 ^a^	32.04 ± 0.25 ^g^	59.78 ± 2.24 ^ab^	38.13 ± 2.59 ^a^	0.62 ± 0.07 ^a^	0.73 ± 0.02 ^cd^	42.00 ± 0.60 ^bcde^	0.72 ± 0.02 ^bcde^
No.9	93.91 ± 0.98 ^abcd^	31.91 ± 0.59 ^g^	59.27 ± 2.29 ^ab^	38.60 ± 2.52 ^a^	0.63 ± 0.07 ^a^	0.68 ± 0.02 ^efgh^	42.01 ± 2.94 ^bcde^	0.73 ± 0.09 ^bcde^
No.10	95.31 ± 0.09 ^ab^	33.80 ± 0.17 ^ef^	61.16 ± 0.36 ^a^	38.83 ± 2.00 ^a^	0.64 ± 0.05 ^a^	0.73 ± 0.01 ^cd^	44.70 ± 1.00 ^abcd^	0.81 ± 0.03 ^abcd^
No.11	93.99 ± 0.71 ^abcd^	32.27 ± 0.10 ^g^	59.18 ± 2.29 ^ab^	33.29 ± 1.49 ^cdef^	0.50 ± 0.03 ^bcdef^	0.78 ± 0.03 ^b^	42.74 ± 1.09 ^bcde^	0.75 ± 0.03 ^bcde^
No.12	95.01 ± 0.27 ^ab^	32.28 ± 0.34 ^g^	59.10 ± 0.86 ^ab^	38.71 ± 1.71 ^a^	0.63 ± 0.05 ^a^	0.76 ± 0.02 ^bc^	40.99 ± 3.54 ^de^	0.70 ± 0.10 ^de^
No.13	95.41 ± 0.37 ^a^	32.48 ± 0.41 ^g^	58.14 ± 0.13 ^ab^	36.53 ± 0.40 ^abcd^	0.58 ± 0.01 ^abc^	0.66 ± 0.01 ^fgh^	45.28 ± 0.86 ^abc^	0.83 ± 0.03 ^abc^
No.14	81.31 ± 1.24 ^g^	36.79 ± 0.24 ^a^	45.98 ± 1.36 ^c^	28.68 ± 3.64 ^g^	0.40 ± 0.07 ^f^	0.57 ± 0.02 ^i^	45.53 ± 0.40 ^ab^	0.84 ± 0.01 ^ab^
No.15	94.15 ± 1.08 ^abc^	34.34 ± 0.46 ^cde^	59.70 ± 1.56 ^ab^	31.99 ± 2.01 ^efg^	0.47 ± 0.04 ^def^	0.69 ± 0.01 ^ef^	44.87 ± 0.39 ^abc^	0.81 ± 0.01 ^abc^
No.16	92.04 ± 1.29 ^abcde^	34.37 ± 0.31 ^cde^	59.36 ± 0.55 ^ab^	36.98 ± 2.53 ^abc^	0.59 ± 0.06 ^ab^	0.68 ± 0.04 ^efg^	42.72 ± 0.69 ^bcde^	0.75 ± 0.02 ^bcde^
No.17	93.91 ± 0.01 ^abcd^	35.88 ± 0.39 ^b^	59.93 ± 1.64 ^ab^	31.77 ± 1.10 ^efg^	0.47 ± 0.02 ^def^	0.70 ± 0.02 ^de^	43.74 ± 1.14 ^abcde^	0.78 ± 0.04 ^abcde^
No.18	89.42 ± 0.91 ^def^	35.03 ± 0.42 ^c^	59.02 ± 1.70 ^ab^	30.15 ± 1.57 ^fg^	0.43 ± 0.03 ^ef^	0.65 ± 0.01 ^h^	46.50 ± 1.13 ^a^	0.87 ± 0.04 ^a^

Data represent mean value ± standard error. Values with different superscripts in a column differ significantly (*p* < 0.05).

**Table 3:** Relative quantification of different subunits of gliadin and HMW glutenin fractions of the 18 HRS composite samples.

Sample ID	ω-Gliadins	α/β-Gliadins	γ-Gliadins	dy HMW-GS	by + dx HMW-GS	bx HMW-GS	ax HMW-GS
No.1	12.00 ± 0.53 ^ab^	40.13 ± 1.26 ^bc^	48.81 ± 1.58 ^bc^	4.18 ± 0.08 ^d^	12.84 ± 0.18 ^ghi^	12.54 ± 0.26 ^a^	4.89 ± 0.04 ^efgh^
No.2	11.63 ± 0.64 ^abcd^	40.77 ± 1.41 ^abc^	48.81 ± 1.38 ^bc^	3.25 ± 0.01 ^j^	11.88 ± 0.26 ^k^	10.70 ± 0.18 ^d^	4.74 ± 0.04 ^h^
No.3	12.02 ± 0.21 ^ab^	40.30 ± 2.28 ^bc^	48.78 ± 0.95 ^bc^	4.53 ± 0.04 ^c^	13.93 ± 0.08 ^cd^	8.22 ± 0.03 ^l^	4.93 ± 0.01 ^efgh^
No.4	12.38 ± 0.09 ^a^	39.07 ± 1.65 ^bc^	50.94 ± 2.13 ^ab^	3.36 ± 0.00 ^i^	13.20 ± 0.03 ^ef^	9.50 ± 0.04 ^h^	5.69 ± 0.01 ^ab^
No.5	12.08 ± 0.64 ^ab^	40.85 ± 2.28 ^abc^	48.67 ± 0.66 ^bc^	4.04 ± 0.13 ^f^	13.94 ± 0.47 ^cd^	9.16 ± 0.26 ^ij^	5.31 ± 0.25 ^cd^
No.6	12.28 ± 0.86 ^a^	39.59 ± 1.00 ^bc^	48.94 ± 2.68 ^bc^	4.06 ± 0.04 ^ef^	13.29 ± 0.06 ^e^	8.94 ± 0.05 ^jk^	5.96 ± 0.00 ^a^
No.7	10.45 ± 0.12 ^cde^	42.48 ± 3.01 ^abc^	48.56 ± 0.79 ^bc^	3.74 ± 0.06 ^h^	13.28 ± 0.04 ^e^	7.57 ± 0.04 ^m^	5.37 ± 0.15 ^cd^
No.8	11.88 ± 0.18 ^ab^	37.95 ± 1.75 ^c^	51.00 ± 1.78 ^ab^	3.85 ± 0.02 ^g^	12.60 ± 0.06 ^hij^	10.04 ± 0.06 ^fg^	5.19 ± 0.01 ^de^
No.9	12.01 ± 0.31 ^ab^	38.91 ± 1.92 ^bc^	50.38 ± 0.93 ^ab^	4.19 ± 0.06 ^d^	12.92 ± 0.00 ^fgh^	11.01 ± 0.21 ^c^	5.06 ± 0.12 ^defg^
No.10	11.24 ± 0.16 ^abcd^	40.62 ± 2.14 ^abc^	49.23 ± 1.42 ^abc^	4.12 ± 0.01 ^def^	12.59 ± 0.16 ^ij^	11.59 ± 0.08 ^b^	5.12 ± 0.12 ^def^
No.11	10.86 ± 0.48 ^bcde^	40.17 ± 2.40 ^bc^	49.78 ± 0.78 ^abc^	3.68 ± 0.01 ^h^	12.48 ± 0.06 ^j^	10.54 ± 0.01 ^de^	5.57 ± 0.02 ^bc^
No.12	11.49 ± 0.24 ^abcd^	37.99 ± 2.09 ^c^	51.91 ± 0.11 ^a^	4.16 ± 0.01 ^de^	13.77 ± 0.09 ^d^	9.83 ± 0.06 ^g^	4.76 ± 0.30 ^gh^
No.13	12.31 ± 0.63 ^a^	41.77 ± 5.09 ^abc^	49.85 ± 0.96 ^abc^	4.20 ± 0.06 ^d^	12.78 ± 0.13 ^ghij^	11.87 ± 0.19 ^b^	5.11 ± 0.04 ^def^
No.14	9.59 ± 0.71 ^e^	46.68 ± 5.11 ^a^	47.52 ± 0.47 ^c^	4.72 ± 0.01 ^a^	14.69 ± 0.01 ^b^	9.20 ± 0.01 ^hij^	5.12 ± 0.14 ^def^
No.15	10.32 ± 0.04 ^de^	44.49 ± 5.70 ^ab^	48.95 ± 0.43 ^bc^	4.66 ± 0.00 ^ab^	15.29 ± 0.08 ^a^	8.75 ± 0.28 ^k^	4.95 ± 0.22 ^efgh^
No.16	12.17 ± 0.80 ^a^	41.21 ± 2.37 ^abc^	49.62 ± 0.80 ^abc^	4.13 ± 0.03 ^def^	13.02 ± 0.02 ^efg^	9.09 ± 0.13 ^ij^	4.89 ± 0.13 ^efgh^
No.17	11.73 ± 1.52 ^abc^	43.35 ± 3.97 ^abc^	49.21 ± 0.56 ^abc^	4.61 ± 0.02 ^bc^	14.04 ± 0.15 ^cd^	9.28 ± 0.01 ^hi^	4.84 ± 0.08 ^fgh^
No.18	12.42 ± 0.91 ^a^	43.80 ± 1.14 ^abc^	48.38 ± 1.77 ^bc^	4.53 ± 0.02 ^c^	14.19 ± 0.03 ^c^	10.26 ± 0.13 ^ef^	4.87 ± 0.31 ^fgh^

Data represent mean value ± standard error. Values with different superscripts in a column differ significantly (*p* < 0.05). “ax”, “bx”, “dx”, and “dy” refer to x- and y-type HMW-GSs encoded by the Glu-1 loci on chromosomes 1A, 1B, and 1D, respectively.

**Table 4:** Starch and AX composition of the 18 HRS composite samples.

Sample ID	Amylose %	Amplopectin %	Amylose/Amylopectin Ratio	Arabinose (mg/g)	Xylose (mg/g)	A:X Ratio
No.1	21.39 ± 0.50 ^bc^	78.61 ± 0.50 ^cd^	0.27 ± 0.01 ^bcd^	7.05 ± 0.92 ^fg^	10.40 ± 0.00 ^de^	0.68 ± 0.09 ^ef^
No.2	21.88 ± 0.40 ^bc^	78.12 ± 0.40 ^cd^	0.28 ± 0.01 ^bcd^	10.45 ± 0.78 ^abcd^	12.85 ± 0.92 ^cd^	0.81 ± 0.00 ^abcd^
No.3	22.14 ± 1.03 ^c^	77.86 ± 1.03 ^d^	0.28 ± 0.02 ^bc^	9.90 ± 2.40 ^bcd^	12.10 ± 2.26 ^cd^	0.81 ± 0.05 ^abcd^
No.4	20.44 ± 1.91 ^cde^	79.56 ± 1.91 ^abc^	0.26 ± 0.03 ^def^	10.90 ± 1.27 ^abc^	13.55 ± 0.92 ^bc^	0.80 ± 0.04 ^abcd^
No.5	21.94 ± 0.17 ^de^	80.57 ± 0.17 ^ab^	0.24 ± 0.00 ^ef^	12.05 ± 0.64 ^a^	13.20 ± 0.28 ^bcd^	0.91 ± 0.07 ^a^
No.6	21.65 ± 0.58 ^e^	80.97 ± 0.58 ^a^	0.24 ± 0.01 ^f^	11.65 ± 0.64 ^ab^	13.70 ± 1.41 ^bc^	0.85 ± 0.04 ^ab^
No.7	21.78 ± 0.79 ^bcd^	79.05 ± 0.79 ^bcd^	0.27 ± 0.01 ^bcde^	7.85 ± 0.07 ^efg^	11.90 ± 0.00 ^cd^	0.66 ± 0.01 ^ef^
No.8	21.14 ± 0.60 ^bc^	78.86 ± 0.60 ^cd^	0.27 ± 0.01 ^bcd^	11.05 ± 0.21 ^abc^	13.60 ± 0.42 ^bc^	0.81 ± 0.01 ^abcd^
No.9	20.42 ± 0.10 ^cde^	79.58 ± 0.10 ^abc^	0.26 ± 0.00 ^def^	11.90 ± 0.85 ^ab^	13.25 ± 0.35 ^bcd^	0.90 ± 0.04 ^a^
No.10	21.01 ± 0.03 ^bc^	78.99 ± 0.03 ^cd^	0.27 ± 0.00 ^bcde^	9.10 ± 0.57 ^cde^	11.40 ± 0.28 ^cde^	0.80 ± 0.07 ^abcd^
No.11	20.59 ± 0.37 ^cd^	79.41 ± 0.37 ^bc^	0.26 ± 0.01 ^cdef^	10.70 ± 0.71 ^abcd^	13.05 ± 0.64 ^cd^	0.82 ± 0.01 ^abcd^
No.12	24.48 ± 1.11 ^a^	75.40 ± 1.11 ^e^	0.33 ± 0.02 ^a^	9.30 ± 0.14 ^cde^	11.60 ± 0.57 ^cd^	0.80 ± 0.03 ^abcd^
No.13	24.74 ± 0.86 ^a^	74.27 ± 0.86 ^e^	0.35 ± 0.02 ^a^	10.25 ± 1.06 ^abcd^	13.55 ± 0.64 ^bc^	0.76 ± 0.04 ^bcde^
No.14	22.45 ± 0.57 ^b^	77.55 ± 0.57 ^d^	0.29 ± 0.01 ^b^	10.15 ± 0.21 ^abcd^	12.35 ± 0.07 ^cd^	0.62 ± 0.14 ^cdef^
No.15	21.24 ± 0.58 ^bc^	78.76 ± 0.58 ^cd^	0.27 ± 0.01 ^bcd^	8.75 ± 1.63 ^def^	12.39 ± 0.69 ^cd^	0.70 ± 0.04 ^f^
No.16	21.29 ± 0.59 ^bc^	78.71 ± 0.59 ^cd^	0.27 ± 0.01 ^bcd^	11.45 ± 1.20 ^ab^	16.03 ± 0.47 ^ab^	0.82 ± 0.02 ^def^
No.17	19.44 ± 0.20 ^de^	80.56 ± 0.20 ^ab^	0.24 ± 0.00 ^ef^	10.10 ± 0.71 ^abcd^	16.95 ± 4.88 ^a^	0.70 ± 0.09 ^abc^
No.18	21.79 ± 0.22 ^bc^	78.21 ± 0.22 ^cd^	0.28 ± 0.00 ^bcd^	5.95 ± 0.21 ^g^	8.50 ± 0.14 ^e^	0.71 ± 0.05 ^cdef^

Data represent mean value ± standard error. Values with different superscripts in a column differ significantly (*p* < 0.05).

**Table 5:** Baking performance of the 18 HRS composite samples.

Sample ID	Baking Absorption (%)	Mix Time (min)	Loaf Volume (mL)	Grain and Texture	Crumb Color	Symmetry
No.1	68.25 ± 0.78 ^f^	4.38 ± 0.18 ^a^	945.00 ± 28.28 ^fg^	7.50 ± 0 ^abc^	8.00 ± 0 ^ab^	9.00 ± 1.41 ^abc^
No.2	66.20 ± 0.71 ^gh^	3.88 ± 0.18 ^cde^	982.50 ± 14.14 ^cdef^	8.00 ± 0 ^ab^	7.65 ± 0.21 ^bc^	9.50 ± 0.71 ^ab^
No.3	70.40 ± 0.71 ^cde^	3.63 ± 0.18 ^ef^	900.00 ± 45.96 ^g^	7.25 ± 0.35 ^bc^	7.15 ± 0.21 ^d^	9.50 ± 0.71 ^ab^
No.4	69.50 ± 0.71 ^e^	3.88 ± 0.18 ^cde^	957.50 ± 49.50 ^def^	7.50 ± 0.71 ^abc^	7.65 ± 0.21 ^bc^	9.00 ± 0.00 ^abc^
No.5	69.50 ± 0 ^e^	3.63 ± 0.18 ^ef^	840.00 ± 21.21 ^h^	7.00 ± 0.71 ^c^	7.75 ± 0.35 ^bc^	7.50 ± 0.71 ^d^
No.6	69.60 ± 0 ^de^	4.00 ± 0 ^bcd^	900.00 ± 42.43 ^g^	7.50 ± 0.71 ^abc^	7.50 ± 0 ^cd^	8.00 ± 0.00 ^cd^
No.7	71.80 ± 0.71 ^ab^	3.13 ± 0.18 ^g^	987.50 ± 24.75 ^bcdef^	7.25 ± 0.35 ^bc^	7.50 ± 0 ^cd^	10.00 ± 0.00 ^a^
No.8	70.70 ± 0.71 ^bcd^	4.25 ± 0 ^ab^	1000.00 ± 31.82 ^bcde^	7.50 ± 0 ^abc^	7.50 ± 0 ^cd^	9.50 ± 0.71 ^ab^
No.9	71.00 ± 0 ^abc^	4.38 ± 0.18 ^a^	1002.50 ± 38.89 ^bcde^	7.75 ± 0.35 ^abc^	8.25 ± 0.35 ^a^	9.00 ± 0.00 ^abc^
No.10	65.80 ± 0.71 ^h^	4.25 ± 0 ^ab^	1010.00 ± 10.61 ^abcd^	8.25 ± 0.35 ^a^	8.00 ± 0 ^ab^	9.00 ± 0.00 ^abc^
No.11	71.40 ± 0.71 ^abc^	4.00 ± 0 ^bcd^	1040.00 ± 10.61 ^ab^	7.50 ± 0 ^abc^	7.15 ± 0.21 ^d^	9.50 ± 0.71 ^ab^
No.12	69.80 ± 0 ^de^	3.63 ± 0.18 ^ef^	1062.50 ± 7.07 ^a^	8.00 ± 0.71 ^ab^	8.00 ± 0 ^ab^	10.00 ± 0.00 ^a^
No.13	66.10 ± 0 ^gh^	4.13 ± 0.18 ^abc^	1012.50 ± 24.75 ^abc^	7.00 ± 0 ^c^	7.50 ± 0 ^cd^	10.00 ± 0.00 ^a^
No.14	71.50 ± 0.71 ^abc^	3.75 ± 0 ^de^	957.50 ± 70.71 ^def^	7.75 ± 0.35 ^abc^	7.65 ± 0.21 ^bc^	9.50 ± 0.71 ^ab^
No.15	71.90 ± 0 ^a^	3.63 ± 0.18 ^ef^	1010.00 ± 24.75 ^bcd^	7.75 ± 0.35 ^abc^	8.00 ± 0 ^ab^	10.00 ± 0.00 ^a^
No.16	68.20 ± 0.71 ^f^	3.38 ± 0.18 ^fg^	955.00 ± 24.75 ^ef^	7.75 ± 0.35 ^abc^	7.65 ± 0.21 ^bc^	9.00 ± 0.00 ^abc^
No.17	67.10 ± 0.71 ^fg^	3.38 ± 0.18 ^fg^	982.50 ± 17.68 ^cdef^	7.00 ± 0.71 ^c^	7.65 ± 0.21 ^bc^	8.50 ± 0.71 ^bcd^
No.18	66.30 ± 0 ^gh^	3.25 ± 0 ^g^	977.50 ± 17.68 ^cdef^	8.00 ± 0 ^ab^	8.15 ± 0.21 ^a^	9.00 ± 0.00 ^abc^

Data represent mean value ± standard error. Values with different superscripts in a column differ significantly (*p* < 0.05).

**Table 6:** Prediction of loaf volume using different parameter combinations.

Components Used for Prediction	Parameters Used for Prediction	R Square	RASE
Protein quantity	GPC at 14%, wet gluten	0.667	31.77
Starch composition	Total starch%, starch damage%, amylose%, amylopectin%, AM/AP	0.634	33.34
Dietary fiber composition	Arabinose, xylose, total arabinoxylans, ara/xyl	0.711	29.62
Protein quantity + starch composition	GPC at 14%, wet gluten, total starch%, starch damage%, amylose%, amylopectin%, AM/AP	0.785	25.55
Protein quantity + dietary fiber composition	GPC at 14%, wet gluten, arabinose, xylose, total arabinoxylans, ara/xyl	0.869	19.9
Starch composition + dietary fiber composition	Total starch%, starch damage%, amylose%, amylopectin%, AM/AP, arabinose, xylose, total arabinoxylans, ara/xyl	0.769	26.48
Protein quantity + starch composition + dietary fiber composition	GPC at 14%, wet gluten, total starch%, starch damage%, amylose%, amylopectin%, AM/AP, arabinose, xylose, total arabinoxylans, ara/xyl	0.862	20.5
SE-HPLC protein composition	UPP%, polymeric%, poly/mono, Glu/Gli	0.733	28.44
Protein quantity + SE-HPLC protein composition	GPC at 14%, wet gluten, UPP%, polymeric%, poly/mono, Glu/Gli	0.831	22.66
RP-HPLC protein composition	HMW-ax, HMW-bx, HMW-by/dx, HMW-dy, ω-gliadins, α/β-gliadins, γ-gliadins, HMW%, HMW/LMW	0.842	21.91
Protein quantity + RP-HPLC protein composition	GPC at 14%, wet gluten, HMW-ax, HMW-bx, HMW-by/dx, HMW-dy, ω-gliadins, α/β-gliadins, γ-gliadins, HMW%, HMW/LMW	0.861	20.55

## Data Availability

Data will be made available on request.

## Supplementary Materials

The following supporting information can be downloaded at https://www.mdpi.com/article/10.3390/foods15040650/s1; Table S1: Pasting properties of the 18 HRS composite samples analyzed by rapid viscosity analyzer (RVA); Table S2: Gluten strength and aggregation properties of the 18 HRS composite samples analyzed by Glutopeak; Table S3: Water absorption capacity and dough mixing properties measured by Farinograph of the 18 HRS composite samples; Table S4: Dough strength and stretching properties of the 18 HRS composite samples measured by Extensograph and Alveograph; Table S5: Correlation analysis between the protein, starch, and fiber compositional factors and flour pasting performance; Table S6: Prediction of loaf volume using simplified linear regression models.

## Abbreviations

The following abbreviations are used in this manuscript:

HMW-GS	High-molecular-weight glutenin subunit
LMW-GS	Low-molecular-weight glutenin subunit
Glu/Gli	Glutenin-to-gliadin ratio
UPP	Unextractable polymeric proteins
EPP	Extractable polymeric proteins
AM/AP	Amylose-to-amylopectin ratio
GPC	Grain protein content
HRS	Hard red spring
SE-HPLC	Size-exclusion high-performance liquid chromatography
PVDF	Polyvinylidene fluoride
RP-HPLC	Reverse-phase high-performance liquid chromatography
WAC	Water absorption capacity
MTI	Mixing tolerance index
LSD	Least significant difference
AX	Arabinoxylan
RVA	Rapid viscosity analyzer
VIP	Variable importance in projection
