# Supplementary material for: Contribution of Protein, Starch, and Fiber Composition to the Prediction of Dough Rheology and Baking Quality in U.S. Hard Red Spring Wheat
Source: Foods. 2026 Feb 11;15(4):650. doi: 10.3390/foods15040650 (PMC12939949; doi:10.3390/foods15040650)
Supplement: Supplementary file 1 [file foods-15-00650-s001.zip › foods-4117485-supplementary.pdf]

Table S1 Pasting properties of the 18 HRS composite samples analyzed by rapid viscosity analyzer (RVA).

**Samples ID**	**Peak Viscosity (cP)**	**Breakdown (cP)**	**Water ± Breakdown (cP)**	**Setback (cP)**	**Peak time (min)**	**Pasting Temperature (℃)**
**No.1**	1949.00 ± 25.46 h	425.00 ± 73.54 bcde	2415.00 ± 19.80 g	891.00 ± 28.28 bcd	6.90 ± 0.14 abc	93.28 ± 1.73 abc
**No.2**	2031.50 ± 6.36 g	337.50 ± 116.67 cde	2538.50 ± 26.16 ef	844.50 ± 15.84 cdef	6.80 ± 0.19 abc	90.88 ± 0.53 bc
**No.3**	2277.50 ± 30.41 a	687.00 ± 41.a	2694.50 ± 13.44 ab	1104.00 ± 04.24 a	7.00 ± 0 abc	70.53 ± 0.60 d
**No.4**	2149.50 ± 38.89 def	330.00 ± 42.de	2512.50 ± 24.75 f	693.00 ± 28.28 efg	6.83 ± 0.05 abc	70.10 ± 0.07 d
**No.5**	1432.50 ± 12.02 j	383.50 ± 47.cde	1570.00 ± 14.14 i	521.00 ± 21.21 g	6.20 ± 0 d	70.58 ± 0.67 d
**No.6**	2312.50 ± 27.58 a	613.50 ± 84.ab	2724.50 ± 23.33 ab	1025.50 ± 39.88 ab	6.97 ± 0.05 ab	71.00 ± 0 d
**No.7**	2130.50 ± 9.19 ef	445.00 ± 107.48 bcde	2581.00 ± 4.24 cde	895.50 ± 53.102 bcd	6.83 ± 0.05 abc	91.68 ± 0.46 abc
**No.8**	2203.50 ± 7.78 cd	454.00 ± 55.15 bcde	2638.50 ± 20.51 bc	889.00 ± 43.42 bcd	6.80 ± 0 abc	92.45 ± 1.70 abc
**No.9**	2163.50 ± 7.78 cde	377.50 ± 61.52 cde	2684.50 ± 12.02 ab	898.50 ± 28.57 bcd	6.77 ± 0.05 bc	92.03 ± 3.36 abc
**No.10**	2025.00 ± 15.56 g	445.50 ± 156.27 bcde	2529.00 ± 12.73 ef	949.50 ± 44.153 abcd	6.87 ± 0.09 abc	94.48 ± 0.04 a
**No.11**	2109.50 ± 7.78 ef	421.50 ± 79.90 bcde	2602.50 ± 2.12 cde	914.50 ± 25.74 bcd	6.87 ± 0.09 abc	94.13 ± 0.46 a
**No.12**	2034.00 ± 5.66 g	449.50 ± 3.bcde	2506.00 ± 22.63 f	921.50 ± 82.31 bcd	6.87 ± 0 abc	92.95 ± 2.26 abc
**No.13**	2097.50 ± 2.12 f	416.50 ± 169.00 bcde	2549.00 ± 52.33 def	868.00 ± 79.118 bcde	6.83 ± 0.14 abc	92.10 ± 3.32 abc
**No.14**	2194.00 ± 18.38 cd	413.00 ± 29.bcde	2635.00 ± 28.28 bc	854.00 ± 60.39 bcdef	6.87 ± 0 abc	68.98 ± 0.53 d
**No.15**	2267.50 ± 58.69 ab	536.00 ± 39.abc	2694.00 ± 33.94 ab	962.50 ± 35.64 abcd	6.97 ± 0.05 ab	70.20 ± 0.07 d
**No.16**	2217.00 ± 15.56 bc	507.00 ± 243.24 abcd	2718.50 ± 60.10 ab	1008.50 ± 70.198 abcd	6.83 ± 0.24 abc	93.70 ± 1.13 ab
**No.17**	1850.00 ± 32.53 i	286.00 ± 29.e	2352.00 ± 43.84 h	788.00 ± 67.46 def	6.70 ± 0.14 c	94.13 ± 0.60 a
**No.18**	1962.00 ± 53.74 h	280.00 ± 28.e	2372.50 ± 24.75 gh	690.50 ± 28.57 fg	6.70 ± 0.05 c	90.50 ± 1.13 c

Data represent mean value ± standard error. Values with different superscripts in a column differ significantly ($P < 0.05$)

Table S2 Gluten strength and aggregation properties of the 18 HRS composite samples analyzed by Glutopeak.

**Sample ID**	**Peak maximum time (min)**	**Torque maximum (BU)**	**Torque before maximum (BU)**	**Torque after maximum (BU)**	**Start-up energy (BEU)**	**Plateau energy (BEU)**	**Aggregation energy (BEU)**
**No.1**	124.33 ± 7.51 abc	56.67 ± 1.15 bcde	52.00 ± 1.73 abc	45.33 ± 0.58 bcd	359.20 ± 36.99 b	303.40 ± 230.64 c	1546.25 ± 26.13 cdef
**No.2**	99.67 ± 7.64 de	56.67 ± 1.15 bcde	52.33 ± 0.58 abc	44.00 ± 0 cd	302.45 ± 84.13 b	121.47 ± 37.40 c	1525.33 ± 2.66 def
**No.3**	84.00 ± 3.46 ef	58.33 ± 2.08 abcd	53.33 ± 3.06 ab	48.33 ± 2.08 ab	952.60 ± 1225.76 ab	230.57 ± 131.91 c	1634.78 ± 24.15 ab
**No.4**	92.33 ± 7.37 def	56.33 ± 1.53 bcde	51.67 ± 2.52 bcd	46.00 ± 3.46 abcd	181.27 ± 15.06 b	177.57 ± 133.38 c	1553.00 ± 40.94 cde
**No.5**	87.00 ± 3.00 ef	55.00 ± 1.00 de	48.33 ± 3.21 d	46.00 ± 2.00 abcd	199.93 ± 66.86 b	123.83 ± 75.24 c	1546.15 ± 34.73 cdef
**No.6**	110.00 ± 2.65 cd	55.00 ± 1.00 de	50.67 ± 1.15 bcd	45.67 ± 0.58 bcd	162.35 ± 12.42 b	459.62 ± 77.64 bc	1524.12 ± 7.70 def
**No.7**	84.67 ± 0.58 ef	59.67 ± 2.52 ab	51.33 ± 2.52 bcd	47.67 ± 0.58 abc	944.12 ± 1054.96 ab	218.18 ± 243.61 c	1617.20 ± 15.38 abc
**No.8**	135.33 ± 5.13 ab	55.00 ± 0 de	51.67 ± 0.58 bcd	44.00 ± 1.00 cd	179.27 ± 26.41 b	641.83 ± 200.45 bc	1506.65 ± 10.85 def
**No.9**	138.33 ± 6.11 a	54.33 ± 1.53 e	50.67 ± 0.58 bcd	44.33 ± 0.58 cd	165.90 ± 17.73 b	769.30 ± 238.66 bc	1493.55 ± 25.62 ef
**No.10**	119.67 ± 10.12 bc	55.33 ± 0.58 de	50.67 ± 0.58 bcd	45.00 ± 1.00 bcd	167.12 ± 17.95 b	392.30 ± 53.23 bc	1519.07 ± 18.03 def
**No.11**	90.33 ± 9.29 ef	56.00 ± 1.00 cde	51.33 ± 2.52 bcd	48.33 ± 4.51 ab	842.65 ± 1139.43 ab	244.30 ± 222.95 c	1569.95 ± 34.85 bcde
**No.12**	122.33 ± 7.57 abc	54.67 ± 0.58 e	51.33 ± 1.15 bcd	44.33 ± 0.58 cd	222.18 ± 116.47 b	575.28 ± 106.24 bc	1507.30 ± 16.26 def
**No.13**	121.33 ± 13.20 abc	55.33 ± 2.08 de	49.67 ± 3.21 cd	47.00 ± 3.46 abcd	213.33 ± 30.54 b	717.08 ± 181.44 bc	1534.08 ± 24.65 def
**No.14**	81.00 ± 20.81 f	60.67 ± 5.03 a	55.33 ± 4.16 a	49.67 ± 3.06 a	1630.40 ± 1256.88 a	1094.78 ± 1381.10 b	1661.18 ± 116.73 a
**No.15**	90.33 ± 19.73 ef	59.00 ± 4.58 abc	50.33 ± 1.53 bcd	46.33 ± 3.79 abcd	1851.38 ± 1456.28 a	1943.28 ± 1297.31 a	1575.82 ± 112.30 bcd
**No.16**	123.00 ± 5.29 abc	54.00 ± 2.65 e	49.67 ± 2.08 cd	43.33 ± 1.53 d	210.23 ± 3.78 b	521.07 ± 222.39 bc	1468.83 ± 65.58 f
**No.17**	84.67 ± 7.09 ef	56.33 ± 1.53 bcde	53.00 ± 0 abc	45.67 ± 0.58 bcd	744.57 ± 717.17 ab	147.40 ± 48.50 c	1547.63 ± 25.56 cde
**No.18**	90.33 ± 22.55 ef	54.67 ± 1.15 e	48.33 ± 1.15 d	46.00 ± 3.61 abcd	347.00 ± 177.34 b	160.15 ± 138.41 c	1521.67 ± 38.17 def

Data represent mean value ± standard error. Values with different superscripts in a column differ significantly (P < 0.05)

Table S3 Water absorption capacity and dough mixing properties measured by Farinograph of the 18 HRS composite samples.

Sample ID	Water absorption capacity (%)	Peak time (min)	Stability (min)	MTI (BU)	FQN (min)
**No.1**	62.85 ± 0.07 f	7.25 ± 0.49 bc	15.35 ± 3.89 abc	19.50 ± 12.02 abcd	167.50 ± 19.09 abcde
**No.2**	62.70 ± 0.14 fg	8.40 ± 0.00 abc	15.10 ± 1.70 abc	18.00 ± 5.66 bcd	173.50 ± 3.54 abcd
**No.3**	63.65 ± 0.07 de	8.45 ± 0.35 abc	14.30 ± 1.70 abcd	14.50 ± 2.12 d	186.50 ± 7.78 abcd
**No.4**	63.55 ± 0.35 e	7.80 ± 0.28 abc	11.75 ± 1.48 cd	22.00 ± 2.83 abcd	148.00 ± 5.66 cde
**No.5**	62.10 ± 0.14 hi	6.95 ± 0.78 c	10.10 ± 0.99 cd	29.00 ± 4.24 ab	73.50 ± 67.18 f
**No.6**	61.70 ± 0.14 ij	8.15 ± 1.20 abc	14.45 ± 2.90 abcd	17.50 ± 2.12 cd	167.50 ± 9.19 abcde
**No.7**	64.50 ± 0.14 bc	8.65 ± 0.07 abc	13.90 ± 1.41 abcd	17.50 ± 6.36 cd	175.00 ± 8.49 abcd
**No.8**	61.80 ± 0.14 ij	9.60 ± 1.98 a	18.95 ± 5.87 ab	21.00 ± 2.83 abcd	202.50 ± 31.82 a
**No.9**	61.70 ± 0.14 ij	8.55 ± 1.63 abc	19.20 ± 6.51 a	17.50 ± 7.78 cd	201.00 ± 36.77 ab
**No.10**	63.45 ± 0.49 e	7.75 ± 0.35 abc	15.05 ± 1.77 abc	20.50 ± 4.95 abcd	167.00 ± 18.38 abcde
**No.11**	63.35 ± 0.07 e	7.55 ± 0.64 abc	12.60 ± 1.84 cd	26.00 ± 4.24 abc	141.00 ± 5.66 de
**No.12**	63.35 ± 0.35 e	8.40 ± 2.12 abc	13.10 ± 2.55 bcd	24.50 ± 2.12 abcd	157.50 ± 20.51 abcde
**No.13**	62.35 ± 0.21 gh	7.25 ± 0.35 bc	15.85 ± 4.17 abc	14.00 ± 7.07 d	185.00 ± 32.53 abcd
**No.14**	64.75 ± 0.35 ab	9.25 ± 0.49 ab	11.50 ± 0.99 cd	24.00 ± 5.66 abcd	162.00 ± 1.41 abcde
**No.15**	65.15 ± 0.21 a	9.35 ± 1.20 ab	14.85 ± 2.19 abc	18.00 ± 1.41 bcd	192.00 ± 21.21 abc
**No.16**	61.35 ± 0.07 j	7.90 ± 1.84 abc	12.55 ± 1.63 cd	23.00 ± 2.83 abcd	152.50 ± 6.36 bcde
**No.17**	64.10 ± 0.00 cd	7.90 ± 1.41 abc	10.20 ± 0.28 cd	23.00 ± 1.41 abcd	143.50 ± 7.78 cde
**No.18**	63.70 ± 0.00 de	6.80 ± 0.42 c	8.55 ± 0.35 d	29.50 ± 6.36 a	122.00 ± 1.41 ef

Data represent mean value ± standard error. Values with different superscripts in a column differ significantly ($P < 0.05$)

Table S4 Dough strength and stretching properties measured by Extensograph and Alveograph of the 18 HRS composite samples.

Sample ID	Extensograph Analyses				Alveograph Analyses			
	Extension 45 (cm)	Resistance 45 (U)	Extension 135 (cm)	Resistance 135 (U)	P (mm H_2O)	L (mm)	W (10^{-4} J)	P/L
**No.1**	16.10 ± 0.85 ab	692.25 ± 2.47 a	11.80 ± 3.25 g	1366.50 ± 126.57 a	108.00 ± 3.00 a	121.00 ± 7.55 fgh	480.67 ± 15.57 a	0.90 ± 0.07 bcd
**No.2**	16.70 ± 0.85 ab	644.75 ± 27.22 ab	12.98 ± 0.53 ef	1152.00 ± 16.97 bc	98.00 ± 2.65 bc	117.67 ± 1.53 ghi	415.00 ± 13.53 b	0.83 ± 0.03 def
**No.3**	16.23 ± 0.60 ab	532.75 ± 6.72 cde	14.20 ± 4.10 fg	1131.00 ± 76.37 bcd	92.00 ± 1.00 ef	126.33 ± 9.45 efg	402.00 ± 21.66 bcde	0.73 ± 0.06 hij
**No.4**	16.78 ± 0.25 ab	543.00 ± 11.31 cd	15.90 ± 1.27 abcd	913.00 ± 117.38 efg	92.33 ± 0.58 ef	114.67 ± 1.53 hi	370.67 ± 5.13 cdef	0.80 ± 0.01 efg
**No.5**	18.48 ± 0.74 a	461.25 ± 3.89 ef	15.95 ± 2.90 abcdef	706.75 ± 54.80 h	72.67 ± 1.15 j	141.67 ± 7.51 abc	319.33 ± 6.81 h	0.51 ± 0.04 k
**No.6**	17.83 ± 0.04 ab	571.50 ± 17.68 bc	16.63 ± 0.18 a	932.75 ± 83.09 efg	80.67 ± 1.53 h	149.00 ± 9.85 a	406.33 ± 25.72 bcd	0.54 ± 0.03 k
**No.7**	17.55 ± 0.92 ab	483.00 ± 57.98 def	16.55 ± 0.64 ab	839.00 ± 77.78 fgh	106.67 ± 0.58 a	113.67 ± 2.52 hi	399.00 ± 4.36 bcde	0.94 ± 0.03 ab
**No.8**	17.20 ± 1.27 ab	698.50 ± 36.06 a	14.40 ± 0.99 abcdef	1177.50 ± 126.57 abc	96.00 ± 1.73 cd	123.00 ± 2.65 efgh	429.00 ± 12.12 b	0.78 ± 0.02 fgh
**No.9**	17.10 ± 0.57 ab	702.00 ± 29.70 a	13.75 ± 0.21 bcdef	1183.50 ± 81.32 abc	93.00 ± 2.65 def	122.33 ± 4.04 efgh	413.00 ± 21.00 bc	0.76 ± 0.03 ghi
**No.10**	17.15 ± 1.48 ab	624.50 ± 38.89 ab	15.70 ± 0.85 abcde	1060.50 ± 156.27 bcde	100.33 ± 3.21 b	109.00 ± 6.24 i	407.67 ± 19.86 bc	0.92 ± 0.06 abc
**No.11**	16.30 ± 1.98 ab	638.00 ± 2.83 ab	14.55 ± 2.47 abcdef	1073.50 ± 2.12 bcde	107.33 ± 2.52 a	109.00 ± 6.08 i	418.67 ± 7.77 b	0.99 ± 0.08 a
**No.12**	17.35 ± 0.07 ab	543.00 ± 24.04 cd	14.75 ± 2.90 defg	911.00 ± 35.36 efg	90.00 ± 1.00 f	120.67 ± 0.58 fgh	380.33 ± 9.29 cdef	0.75 ± 0.01 ghi
**No.13**	15.40 ± 0.99 b	682.50 ± 111.02 a	13.45 ± 1.20 def	1233.50 ± 92.63 ab	99.00 ± 4.36 bc	115.67 ± 14.57 hi	419.67 ± 60.38 b	0.86 ± 0.08 cde
**No.14**	16.75 ± 0.07 ab	489.50 ± 13.44 de	16.20 ± 3.54 abcdef	917.50 ± 10.61 efg	86.00 ± 0 g	129.67 ± 2.08 def	373.33 ± 4.16 def	0.66 ± 0.01 j
**No.15**	17.30 ± 2.40 ab	492.00 ± 15.56 de	14.60 ± 3.39 fg	956.00 ± 21.21 def	94.33 ± 3.06 de	132.33 ± 4.04 cde	426.67 ± 16.07 b	0.71 ± 0.03 ij

**No.16**	16.45 ± 0.49 ab	516.00 ± 52.33 cde	14.50 ± 2.40 abcdef	1002.50 ± 13.44 cdef	76.00 ± 1.00 ij	141.67 ± 5.77 abc	354.00 ± 10.44 fg	0.54 ± 0.02 k
**No.17**	16.65 ± 0.07 ab	516.50 ± 10.61 cde	15.85 ± 4.03 cdefg	899.00 ± 72.12 efgh	79.33 ± 2.08 hi	137.00 ± 5.00 bcd	353.00 ± 14.80 fg	0.58 ± 0.03 k
**No.18**	16.90 ± 2.26 ab	405.50 ± 9.19 f	16.40 ± 0.85 abc	759.50 ± 198.70 gh	77.33 ± 2.08 hi	144.00 ± 3.00 ab	337.00 ± 15.10 gh	0.54 ± 0.02 k

Data represent mean value ± standard error. Values with different superscripts in a column differ significantly ($P < 0.05$)

Table S5 Correlation analyses between different traits.

	**Peak maximum time**	**Torque maximum**	**Torque before maximum**	**Torque after maximum**	**Start-up energy**	**Plateau energy**	**Aggregation energy**	**WAC**	**Peak Time**
**GPC at 14**	-0.505**	0.325	0.401*	0.259	0.568**	0.329	0.402*	0.416*	0.326
**Ash%**	-0.674**	0.344*	0.19	0.426**	0.513**	0.165	0.435**	0.570**	0.134
**Glu Index**	0.581**	-0.387*	-0.174	-0.348*	-0.261	-0.003	-0.490**	-0.242	-0.2
**Wet Gluten%**	-0.580**	0.362*	0.27	0.341*	0.408*	0.106	0.491**	0.422*	0.174
**ω-gliadin**	0.362*	-0.577**	-0.272	-0.340*	-0.534**	-0.380*	-0.512**	-0.600**	-0.632**
**α/β-gliadin**	-0.476**	0.545**	0.390*	0.264	0.640**	0.389*	0.509**	0.441**	0.115
**γ-gliadin**	0.446**	-0.374*	-0.169	-0.291	-0.318	-0.09	-0.431**	-0.223	-0.19
**HMW-dy**	-0.248	0.199	0.139	0.255	0.560**	0.435**	0.284	0.342*	0.101
**HMW-by/dx**	-0.623**	0.460**	0.144	0.390*	0.727**	0.514**	0.537**	0.596**	0.292
**HMW-bx**	0.523**	-0.369*	-0.126	-0.17	-0.28	-0.143	-0.354*	-0.324	-0.381*
**HMW-ax**	-0.032	-0.016	-0.07	0.138	-0.186	-0.096	0.103	-0.167	-0.028
**HMW%**	-0.177	0.223	0.094	0.331*	0.419*	0.27	0.344*	0.278	-0.057
**HMW/LMW**	-0.17	0.225	0.11	0.327	0.418*	0.256	0.342*	0.277	-0.059
**UPP%**	0.339*	-0.485**	-0.473**	-0.458**	-0.463**	-0.295	-0.581**	-0.251	-0.401*
**Poly%**	0.791**	-0.508**	-0.194	-0.405*	-0.483**	-0.056	-0.518**	-0.562**	-0.351
**Poly/Mono**	0.794**	-0.506**	-0.186	-0.408*	-0.469**	-0.053	-0.519**	-0.549**	-0.346
**Glutenin/Gliadin**	0.292	-0.168	-0.103	-0.215	-0.437**	-0.366*	-0.231	-0.139	-0.093
**Starch Damage**	-0.112	0.069	-0.350*	0.248	0.091	0.099	0.127	0.350*	0.016
**Total Starch%**	0.303	-0.261	-0.188	-0.414*	-0.233	-0.073	-0.421*	-0.288	-0.365*
**Amylose %**	0.093	0.077	0.028	0.19	0.043	0.178	0.125	0.092	0.008
**Amylopectin%**	-0.093	-0.077	-0.028	-0.19	-0.043	-0.178	-0.125	-0.092	-0.008
**AM/AP**	0.097	0.071	0.028	0.185	0.037	0.173	0.119	0.084	-0.004
**Arabinose%**	0.261	-0.269	0.134	-0.217	-0.15	0	-0.294	-0.526**	-0.067
**Xylose%**	0.128	-0.177	0.104	-0.189	0.068	0.015	-0.258	-0.269	-0.058
**Total Arabinoxylans%**	-0.296	0.314	0.349*	0.344*	0.421*	0.384*	0.400*	0.175	0.379*
**Ara/xyl**	0.208	-0.378*	0.027	-0.181	-0.446**	-0.336*	-0.308	-0.483**	-0.224

	Stablity	MTI	FQN	Extension 45	Resistance 45	Extension 135	Resistance 135	P	L
**GPC at 14**	-0.225	-0.093	0.089	-0.083	-0.414*	-0.237	-0.156	-0.426**	0.386*
**Ash%**	-0.492**	0.156	-0.398*	-0.168	-0.546**	0.061	-0.396*	-0.279	0.206
**Glu Index**	0.441*	-0.248	0.247	-0.149	0.590**	-0.117	0.507**	0.421*	-0.316
**Wet Gluten%**	-0.347	0.057	-0.137	-0.008	-0.593**	0.077	-0.350*	-0.417*	0.463**
**ω-gliadin**	-0.206	0.09	-0.242	0.005	0.186	-0.1	0.134	-0.29	0.195
**α/β-gliadin**	-0.392*	0.379*	-0.302	-0.114	-0.484**	-0.272	-0.321	-0.219	0.126
**γ-gliadin**	0.223	-0.322	0.21	-0.078	0.360*	0.198	0.291	0.133	-0.21
**HMW-dy**	-0.335	0.012	-0.004	-0.051	-0.354*	-0.177	-0.128	-0.366*	0.436**
**HMW-by/dx**	-0.502**	0.149	-0.06	0.169	-0.707**	-0.007	-0.527**	-0.458**	0.470**
**HMW-bx**	0.107	-0.072	-0.093	-0.293	0.682**	-0.276	0.612**	0.384*	-0.324
**HMW-ax**	-0.019	-0.038	-0.031	0.068	0.047	0.496**	-0.164	0.022	-0.041
**HMW%**	-0.364	0.033	-0.098	-0.186	-0.071	-0.311	0.122	-0.04	0.179
**HMW/LMW**	-0.361	0.031	-0.097	-0.191	-0.049	-0.316	0.139	-0.02	0.165
**UPP%**	0.052	-0.127	0.011	0.113	0.123	0.297	-0.062	0.077	-0.084
**Poly%**	0.446*	-0.225	0.337	-0.099	0.565**	-0.22	0.462**	0.207	-0.272
**Poly/Mono**	0.454*	-0.234	0.350*	-0.099	0.564**	-0.2	0.463**	0.205	-0.271
**Glutenin/Gliadin**	0.403*	-0.231	0.184	-0.036	0.434**	0.012	0.387*	0.534**	-0.338*
**Starch Damage**	0.009	-0.152	-0.099	-0.158	-0.033	0.291	-0.041	0.279	-0.26
**Total Starch%**	-0.247	0.111	-0.234	-0.102	-0.084	0.1	-0.073	-0.146	0.215
**Amylose %**	0.04	-0.086	0.051	-0.352*	0.129	-0.406*	0.252	0.23	-0.429**
**Amylopectin%**	-0.04	0.086	-0.051	0.352*	-0.129	0.406*	-0.252	-0.23	0.429**
**AM/AP**	0.034	-0.087	0.044	-0.348*	0.129	-0.402*	0.249	0.222	-0.427**
**Arabinose%**	0.124	-0.022	0.059	0.019	0.264	-0.145	0.075	-0.283	0.109
**Xylose%**	0.013	-0.077	0.038	-0.076	0.124	-0.13	0.061	-0.289	0.169
**Total Arabinoxylans%**	-0.094	0.033	0.133	0.083	0.016	-0.071	-0.129	-0.069	0.011
**Ara/xyl**	-0.051	0.211	-0.139	0.328	0.248	-0.024	-0.033	-0.206	0.054

	W	P/L	Baking absorption%	Mix Time	Final Volume	Grain and texture	Crumb Color	Symmetry
**GPC at 14**	-0.24	-0.467**	-0.015	-0.336*	-0.274	-0.128	-0.109	-0.066
**Ash%**	-0.371*	-0.257	-0.231	-0.549**	0.039	-0.12	-0.089	0.028
**Glu Index**	0.496**	0.395*	-0.311	0.397*	0.549**	0.151	0.076	0.217
**Wet Gluten%**	-0.326	-0.474**	0.107	-0.456**	-0.511**	-0.221	-0.123	-0.24
**ω-gliadin**	-0.156	-0.235	-0.483**	0.166	-0.245	-0.097	0.131	-0.231
**α/β-gliadin**	-0.31	-0.18	-0.051	-0.375*	0.01	-0.094	0.124	0.131
**γ-gliadin**	0.125	0.171	0.07	0.292	0.348*	0.061	-0.01	0.029
**HMW-dy**	-0.145	-0.444**	0.06	-0.211	-0.079	-0.04	0.248	-0.03
**HMW-by/dx**	-0.367*	-0.522**	0.353*	-0.490**	-0.183	-0.057	0.203	-0.044
**HMW-bx**	0.465**	0.388*	-0.531**	0.717**	0.305	0.21	0.349*	0.003
**HMW-ax**	-0.027	0.051	0.32	0.166	-0.228	-0.133	-0.341*	-0.293
**HMW%**	0.127	-0.121	-0.104	0.132	-0.048	-0.024	0.341*	-0.041
**HMW/LMW**	0.146	-0.103	-0.123	0.15	-0.047	-0.005	0.350*	-0.032
**UPP%**	-0.001	0.107	-0.203	-0.026	0.11	0.027	0.129	-0.161
**Poly%**	0.273	0.253	-0.125	0.496**	0.091	0.09	0.239	-0.099
**Poly/Mono**	0.272	0.25	-0.118	0.500**	0.099	0.114	0.257	-0.095
**Glutenin/Gliadin**	0.529**	0.472**	-0.04	0.297	0.101	0.055	-0.126	0.05
**Starch Damage**	0.081	0.297	-0.225	0.007	0.302	0.064	0.252	0.288
**Total Starch%**	-0.114	-0.175	-0.118	-0.288	0.016	0.022	0.304	-0.067
**Amylose %**	0.039	0.337*	-0.188	0.02	0.424**	0.036	0.067	0.602**
**Amylopectin%**	-0.039	-0.337*	0.188	-0.02	-0.424**	-0.036	-0.067	-0.602**
**AM/AP**	0.031	0.333*	-0.193	0.025	0.423*	0.023	0.062	0.594**
**Arabinose%**	-0.151	-0.213	0.183	0.218	-0.149	-0.142	-0.289	-0.185
**Xylose%**	-0.186	-0.242	0.007	-0.039	-0.048	-0.183	-0.243	-0.078
**Total Arabinoxylans%**	-0.023	-0.068	0.338*	0.149	0.008	-0.117	-0.045	0.024
**Ara/xyl**	-0.144	-0.141	-0.062	0.285	-0.23	-0.11	-0.134	-0.364*

Table S6 Prediction of loaf volume using simplified linear regression models.

Components used for prediction	Parameters used for prediction	Equations	R Square
**Protein quantity**	GPC at 14%, wet gluten	Final volume = 1382.35 - 21.50 * wet gluten + 20.90 * GPC at 14%	0.6
**Starch composition**	Total starch%, starch damage%, amylose%, amylopectin%, AM/AP	Final volume = -112.5 - 0.86 * Total starch% + 155.27 * amylose% - 8807.28 * AM/AP + 35.55 * starch damage%	0.45
**Dietary fiber composition**	Arabinose, Xylose, Total Arabinoxylans, Ara/Xyl	Final volume = 1000.14 - 9.18 * Arabinose + 5.34 * Xylose + 2.18 * Total Arabinoxylans - 68.72 * Ara/Xyl	0.14
**Protein quantity + Starch composition**	GPC at 14%, wet gluten, Total starch%, starch damage%, amylose%, amylopectin%, AM/AP	Final volume = 105.79 -18.39 * wet gluten - 0.67 * Total starch% + 196.25 * amylose% - 11467.38 * AM/AP + 15.66 * GPC at 14% + 35.79 * starch damage	0.68
**Protein quantity + Dietary fiber composition**	GPC at 14%, wet gluten, Arabinose, Xylose, Total Arabinoxylans, Ara/Xyl	Final volume = 1692.30 + 9.01 * GPC at 14% - 21.94 * wet gluten - 8.29 * Arabinose + 2.30 * Xylose + 1.84 * Total Arabinoxylans - 131.22 * Ara/Xyl	0.56
**Starch composition + Dietary fiber composition**	Total starch%, starch damage%, amylose%, amylopectin%, AM/AP, Arabinose, Xylose, Total Arabinoxylans, Ara/Xyl	Final volume = -671.03 + 34.28 * starch damage% + 0.82 * Total starch% + 221.44 * amylose% - 12702.75 * AM/AP - 6.32* Arabinose + 5.74 * Xyl + 1.42 * Total Arabinoxylans + 73.32 * Ara/Xyl	0.4
**Protein quantity + Starch composition + Dietary fiber composition**	GPC at 14%, wet gluten, Total starch%, starch damage%, amylose%, amylopectin%, AM/AP, Arabinose, Xylose, Total Arabinoxylans, Ara/Xyl	Final volume = 742.93 + 0.86 * GPC at 14% -16.56 * wet gluten + 17.05* starch damage% - 2.15 * Total starch% + 146.64 * amylose% - 8442.43* AM/AP - 6.14 * Arabinose + 3.68 * Xylose + 1.32 * Total Arabinoxylans - 44.06 * Ara/Xyl	0.62
**SE-HPLC Protein composition**	UPP%, Polymeric%, Poly/Mono, Glu/Gli	Final volume = 1118.16 + 1.72 * UPP% - 25.96 * Polymeric% + 1153.01 * Poly/Mono + 53.55 * Glu/Gli	0.09
**Protein quantity + SE-HPLC Protein composition**	GPC at 14%, wet gluten, UPP%, Polymeric%, Poly/Mono, Glu/Gli	Final volume = 2158.07 + 19.32 * GPC at 14% - 24.16 * wet gluten - 0.97 * UPP% - 45.03 * Polymeric% + 1846.47 * Poly/Mono - 42.74 * Glu/Gli	0.53
**RP-HPLC Protein composition**	HMW-ax, HMW-bx, HMW-by/dx, HMW-dy, ω-gliadins, α/β-gliadins, γ-gliadins, HMW%, HMW/LMW	Final volume = -872.36 -22.59 *ω-gliadins + 6.45 *α/β-gliadins + 16.78 *γ-gliadins - 16.48 * HMW-dy - 1.84 * HMW-by/dx + 19.28 * HMW-bx - 23.84 * HMW-ax + 58.55 * HMW% - 1952.42 * HMW/LMW	0.68
**Protein quantity + RP-HPLC Protein composition**	GPC at 14%, wet gluten, HMW-ax, HMW-bx, HMW-by/dx, HMW-dy, ω-gliadins, α/β-gliadins, γ-gliadins, HMW%, HMW/LMW	Final volume = 615.79 - 15.90 * wet gluten + 6.66 * GPC at 14% - 20.85 *ω-gliadins + 5.55 *α/β-gliadins + 12.30 * γ-gliadins + 25.15 * HMW-dy - 10.02 * HMW-by/dx + 6.04 * HMW-bx - 8.87 * HMW-ax + 12.24 * HMW% - 408.09 * HMW/LMW	0.74
